# New insights into neurodevelopmental disorders by whole genome sequencing of 100 families from Italy

**DOI:** 10.1038/s41525-025-00547-8

**Published:** 2026-02-02

**Authors:** Giovanni Spirito, Sara Trova, Gaia Treves, Khudayar Farmanli, Mariacristina Franzese Canonico, Agata Fant, Stefano Marangoni, Federica Furia, Debora Charrance, Nicola Locci, Sara Gottardo, Francesca Groppo, Vittoria Perseghin, Martina Toscano, Erika Bibbò, Serena Donetti Dontin, Cecilia Cargnelutti, Mélody Colliard, Alessandro Rosina, Anna Maria Beoni, Cristina Bérard, Alessandro Coppe, Paolo Serravalle, Fabio Landuzzi, Francesco Musacchia, Antonio Amoroso, Remo Sanges, Andrea Cavalli, Manuela Vecchi, Laure Obino, Stefano Gustincich

**Affiliations:** 1 Non-coding RNAs and RNA-based therapeutics, Italian Institute of Technology, CMP3VdA, Aosta, Italy; 2 Center for Clinical and Computational Genomics, Non-coding RNAs and RNA-based therapeutics, Fondazione Istituto Italiano di Tecnologia (IIT), Aosta, Italy; 3 Computational and Chemical Biology, Italian Institute of Technology, CMP3VdA, Aosta, Italy; 4 Center for Clinical and Computational Genomics, Computational and Chemical Biology, Fondazione Istituto Italiano di Tecnologia (IIT), Aosta, Italy; 5 Pediatric Neuropsychiatry, Healthcare agency of the Aosta Valley, Beauregard Hospital, Aosta, Italy; 6 Psychiatry, Healthcare agency of the Aosta Valley, Aosta, Italy; 7 Pediatrics, Healthcare agency of the Aosta Valley, Beauregard Hospital, Aosta, Italy; 8 Center for Human Technologies, Non-coding RNAs and RNA-based therapeutics, Italian Institute of Technology, Genova, Italy; 9 Immunogenetics and Transplant Biology Service, Città Della Salute e Della Scienza di Torino, University Hospital, Turin, Italy; 10 Area of Neuroscience, International School for Advanced Studies (SISSA), Trieste, Italy; 11 Center for Human Technologies, Computational and Chemical Biology, Fondazione Istituto Italiano di Tecnologia, Genoa, Italy; 12 Centre Européen de Calcul Atomique et Moléculaire (CECAM), Ecole Polytechnique Fédérale de Lausanne, Lausanne, Switzerland

**Keywords:** Diseases, Genetics, Neuroscience

## Abstract

Neurodevelopmental disorders (NDDs) have a strong but largely unexplained genetic basis. Moreover, the genetic architecture of these complex disorders in under-represented communities is poorly studied. We analyzed 110 probands from 100 families (for a total of 298 individuals), using whole genome sequencing (WGS) to identify genetic contributors to NDDs. This study is part of the ‘5000genomi@VdA’ project characterizing Valle d’Aosta (Italy) genomic landscape in health and disease. Probands were stratified into three diagnostic categories: ASD (autism spectrum disorder without intellectual disability), ID (intellectual disability without ASD), and ASD-ID (autism spectrum disorder with comorbid intellectual disability). Following the ACMG guidelines, we identified 32 likely phenotype-causing variants in known NDD-associated genes in 26.4% of the probands. We observed a diagnostic yield gradient, lowest in ASD, intermediate in ASD-ID, and highest in ID. We also identified 42 variants of uncertain significance, 14 of which were located in genes not previously linked to NDDs but relevant to neurodevelopment, and may thus represent new NDD candidate genes. Furthermore, we used Evo 2, an evolutionary constraint-based model, to refine variant interpretation and identify VUS with pathogenic-like signatures. These findings highlight the utility of WGS in exploring the genetic heterogeneity within stratified NDD clinical groups.

## Introduction

Neurodevelopmental disorders (NDDs) encompass a diverse group of conditions that affect the development of the central nervous system (CNS), leading to a wide range of cognitive, motor, and behavioral impairments^1,2^. Among them, Autism Spectrum Disorder (ASD) and Intellectual Disability (ID) are the most prevalent, posing significant challenges for affected individuals, their families, and healthcare systems worldwide^3^.

Although highly heritable^4–8^, with estimates reaching 80-90% for ASD based on twin and sibling studies^9–11^, the genetic basis of many NDDs remains incompletely understood^1,2^, complicating diagnosis and treatment.

The global prevalence of ASD and ID is estimated to be approximately 1%^1^. In Italy, a nationwide study has recently reported a prevalence of 13.4 ASD cases per 1000 children aged 7–9 years^12^, consistent across northern, central, and southern regions. However, a precise mapping of the prevalence of ASD/ID in each region of the country is still missing, and the genetic architecture of these complex disorders in the different areas and under-represented small populations is poorly studied.

Over the past decade, next-generation sequencing (NGS) has transformed the study of NDD genetics^13^. Whole Exome Sequencing (WES) is the most cost-effective method to identify Single-Nucleotide Variants (SNVs) and small insertions and deletions (Indels) of clinical relevance^14–17^. However, the majority of diagnosed individuals, approximately 70%, remain unsolved^18–20^, partly because WES lacks the capability to reliably detect structural variants (SV), copy-number variants (CNV), and non-coding alterations. On the other hand, whole genome sequencing (WGS), though more expensive and computationally demanding, is a comprehensive technology able to identify a broader spectrum of genomic variants that may be missed by conventional methods^13,21,22^. Indeed, recent studies indicate that WGS can enhance diagnostic yield by at least 8% compared to WES^18^. Importantly, multiple studies underline the importance of intronic SVs^23,24^, CNVs^25–27^, Mobile Elements Insertions (MEI)^28,29^, and Tandem-Repeat Expansions^30,31^ in NDDs.

Currently, high-throughput sequencing of large cohorts of NDD families has led to the discovery of thousands of genes involved in both the pathogenesis or susceptibility of these disorders^13,32–34^. Several NDD-related genes are strongly associated with specific phenotypes^32,35^, while others are involved in broader neurodevelopmental processes and are not strictly tied to specific clinical presentations^34^. This latter group, although not immediately adopted for diagnostic utility, may offer insights into the fundamental mechanisms of neurodevelopment and the genetic basis of NDDs^36,37^. Overall, reconstructing the genetic architecture of NDDs is challenging due to their complexity and heterogeneity.

In this study, we sequenced and analyzed whole genomes of 110 probands affected by NDDs (100 families) and their parents (298 individuals in total). We stratified probands in three main clinical categories: ASD (autism spectrum disorder without intellectual disability, 40 cases), ID (intellectual disability without ASD, 27 cases), and ASD-ID (autism spectrum disorder with comorbid intellectual disability and/or severe language impairment, 43 cases). Our investigation examined SNVs, indels, SVs, CNVs, and MEIs, providing a comprehensive view of the genetic landscape underlying these heterogeneous conditions.

This investigation is part of the broader ‘5000genomi@VdA’ research initiative, supported by the Valle d’Aosta regional government, which aims to elucidate the genomic architecture of this population. Our objective is to achieve deep molecular profiling of affected families subdivided into three main clinical groups by identifying both clinically relevant variants in established disease-causing genes and novel candidate genes implicated in NDDs, thereby contributing to the expansion of scientific knowledge and promoting the development of personalized therapeutic strategies for these complex disorders.

## Results

### The study cohort

We recruited 110 probands diagnosed with neurodevelopmental phenotypes comprising ASD, ID, or both (78 males and 32 females) from 100 families (298 individuals in total) (Table 1; Supplementary Data 1 and Table S1). Among the 110 probands, 90 were from simplex families, while 20 belonged to multiplex families (including one pair of dizygotic twins). For 10 probands, only one parent genome was available for sequencing, and for 2 probands, parental genomes could not be sequenced.

**Table 1:** Overview of individuals enrolled in the study

	Probands (n)	Parents (n)	Proband age at enrollment mean (± stdev)
Demographic composition			
Total	110	188	12.3 (±7)
Male	78 (71%)	91 (49%)	11.9 (±6.1)
Female	32 (29%)	97 (51%)	13.3 (±8.8)
Diagnostic group			
ASD	40 (36.5%)	na	16.5 (±6.8)
ID	27 (24.5%)	na	10.8 (±5.3)
ASD-ID	43 (39%)	na	9.5 (±6.2)

We stratified the probands into three main clinical groups: 40 with ASD alone, 27 with ID, and 43 with mixed ASD-ID (Supplementary Data 1 and Table S1). Although we did not observe significant differences in age at diagnosis between male and female probands, we found that probands in the ASD group were, on average, diagnosed at an older age than those in the other two groups (Supplementary Data 1 and Table S1). This likely reflects the predominance of high-functioning cases (67.5%, 27 out of 40) in the ASD group, who are typically diagnosed later in life. Among the ten multiplex families with two probands each, four pairs were assigned to different experimental groups due to marked differences in their clinical phenotypes. Of the remaining six pairs assigned to the same group, two exhibited mild but notable phenotypic differences (Supplementary Data 1 and Table S1). Our cohort did not include unaffected siblings. In 18 families, one or both parents, although not formally diagnosed with an NDD, exhibited behavioral or cognitive traits potentially relevant to the NDD spectrum (Supplementary Data 1 and Table S1). As expected, all parent-proband and sibling-sibling pairs displayed a kinship coefficient of approximately 0.25 (Supplementary Data 1 and Table S2). All mother-father pairs were confirmed to be unrelated (kinship coefficient around 0); with the exception of parents of family NED052, the father (NED052_F) and the mother (NED052_M), who showed a kinship coefficient of 0.03 (Supplementary Data 1 and Table S2), suggestive of potential fourth-degree relatedness. It is important to note that populations with a history of genetic drift, founder effects, or inbreeding, may exhibit slightly elevated background levels of relatedness, which could influence kinship estimates. We performed WGS on all 298 participants, achieving an average coverage of 38X. Detailed quality metrics for *fastq* files and alignment files are provided in Tables S3–S5.

### Overview of short variants and structural variants identified in the cohort

**Fig. 1:**
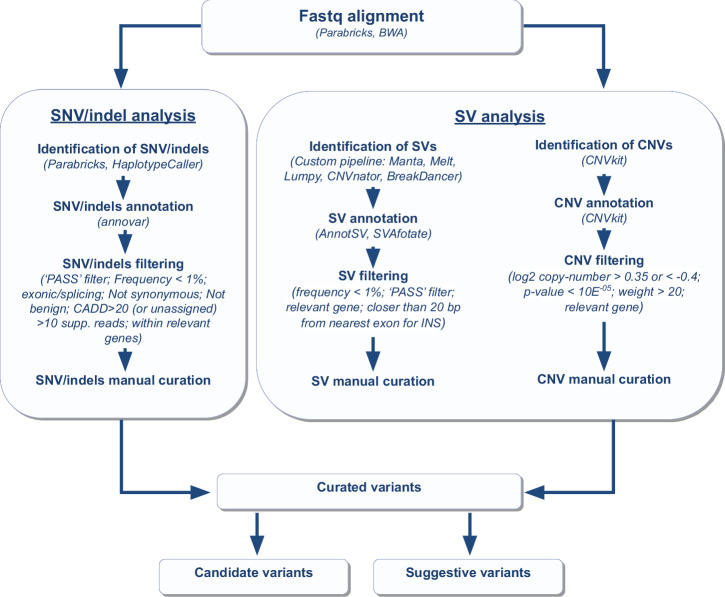
Overview of the methods used for variant discovery and prioritization. Created in microsoft powerpoint.

**Fig. 2:**
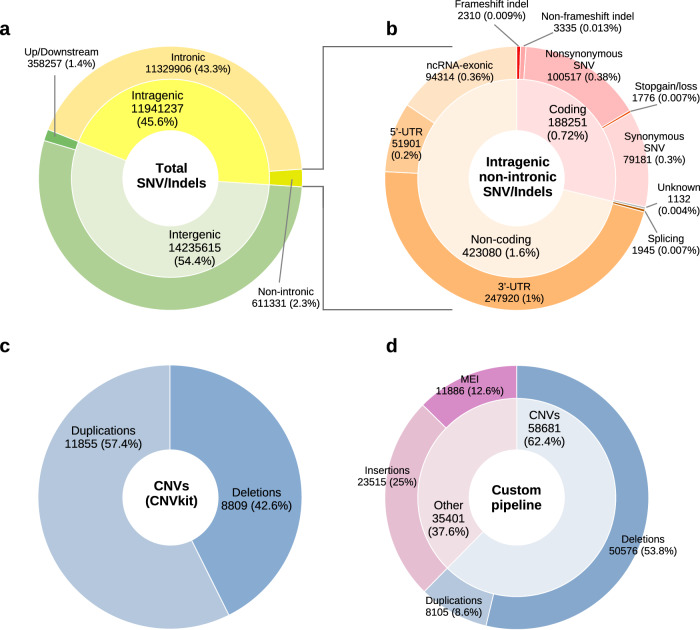
Summary of genomic variants identified in study participants; **a**, **b** percentages of short variants categories are related to the total number of short variants identified, short variants identified with Parabricks were filtered using the following criteria: marked as “PASS,” supported by more than 10 reads, and containing a single genotype. **a** total genomic short variants, **b** intergenic non-intronic short variants; **c** percentages and counts of CNVs detected related to the total number of CNVs identified with CNVkit, CNVs were filtered for a log₂ copy-number ratio > 0.35 or <−0.4, a *p*-value < 1×10^−^^5^; **d** percentages and counts of SVs detected with our custom SV pipeline related to the total number of SVs identified, SVs identified using the custom pipeline were filtered to include only those marked as “PASS”. Created with R (ggplot2 package).

We performed short variant calling (SNVs and indels) across the cohort of 298 individuals (comprising probands and parents) using the NVIDIA Clara™ Parabricks^®^ pipeline (version 3.8) (Fig. 1). This analysis identified a total of 26,176,852 unique variants (21,745,848 SNVs and 4,431,004 indels) (Fig. 2a,b). The variants were then prioritized according to a procedure outlined in the Methods section, with the objective of identifying a subset of potentially causative variants present in the probands that correlate with their phenotypes. After filtering, we identified a total of 3703 short variants across the 110 probands (Supplementary Data 1 and Table S6). Of these, 3334 were heterozygous and inherited from one parent, 148 were heterozygous but could not be definitively classified as de novo or inherited (due to the unavailability of one parental genome) and are thus referred to as “putative de novo” variants, 37 were confirmed as de novo, 33 were X-linked (found in hemizygosity in male probands and inherited maternally) and 5 were homozygous and inherited from both parents. We were unable to clearly determine the segregation pattern for approximately 2.3% of the identified variants (85 variants out of 3703).

Filtered variants were then manually curated to prioritize those that most likely contribute to the probands phenotype. The most significant findings are detailed below (Tables 2–4).

**Table 2:** Candidate and suggestive variants in probands affected by ASD

Proband	Variant	Variant Type	Candidate gene	Segregation	ACMG Class	ClinVar	Sex
Candidate							
NED012_P	NM_017635.5:c.2347C > T(p.Arg783Ter)	Nonsense SNV	*KMT5B*	de novo	P (PVS1, PS6, PS2, PM2)	LP	M
NED092_P	NM_152296.5:c.1790G > A(p.Arg597His)	Missense SNV	*ATP1A3*	de novo	P (PM2, PS2, PP3, PM5, PP2, PP5); AM = LP	VUS	F
NED016_P	NM_006662.3:c.6729+9dup(p.?)	Splicing indel (ins)	*SRCAP*	Inherited (M)	LP (PVS1, PM2)	VUS	M
**NED016_P**	**NM_006662.2:c.2494** **G** > **A(p.Val832Ile)**	**Missense SNV**	***SRCAP***	**Inherited (F)**	**VUS warm (PM2, PP3); AM** = **LP**	**-**	**M**
NED045_P	NC_000015.10:g.22579193_23125407del	Deletion	*NIPA1, NIPA2, TUBGCP5, CYFIP1 (pH* = *0.1;0.4;0.2;0.84)*	Inherited (F)	P (1)	-	F
NED007_P1	NC_000001.11:g.2538784_3205123del	Deletion	*MMEL1, PRDM16, ACTR2 (pH* = *0.37;0.99;0.96)*	de novo	LP (0.9)	-	M
NED082_P	NC_000016.10:g.89640587_89914558del	Deletion	*CDK10, CHMP1A, FANCA*	Inherited (M)	P (1)	-	M
NED060_P	NC_000015.10:g.31714997_32155815dup	Duplication	*CHRNA7, OTUD7A (pT* = *0.58;0.3)*	Inherited (M)	VUS (0.3)	-	M
NED065_P	NM_013275.6:c.-145 + 32621_-60+2372del(p.?)	Deletion	*ANKRD11*	Inherited (M)	VUS (0.3)	-	M
NED086_P	NM_001374828.1:c.588_589insAGCAGCAGCAGCAGCAGCAGCAAC(p.Phe196_Gln197insSerSerSerSerSerSerSerAsn)	Indel (ins)	*ARID1B*	de novo	VUS (PM2, PM4, PM6, BP4)	VUS	M
NED092_P	NM_152641.4:c.3197C > T(p.Pro1066Leu)	Missense SNV	*ARID2*	de novo	VUS (PM2); AM = LP	-	F
**NED055_P**	**NM_004187.5:c.3506C** > **G(p.Ser1169Trp)**	**Missense SNV**	***KDM5C***	**X-linked**	**VUS (PM2, PP2); AM** = **LB**	**-**	**M**
NED020_P2	NM_001368397.1:c.3598 T > C(p.Ser1200Pro)	Missense SNV	*FRMPD4*	X-linked	VUS (PM2, BS2); AM = LB	-	M
**NED049_P1**	**NM_000240.4:c.608G** > **A(p.Gly203Asp)**	**Missense SNV**	***MAOA***	**X-linked**	**VUS (PM2, PP3, BS2); AM** = **LP**	**-**	**M**
NED024_P	NM_001128840.3:c.5935C > T(p.Arg1979Trp)	Missense SNV	*CACNA1D*	Putative de novo	VUS (PM2, BP4); AM = LB	VUS	M
NED024_P	NM_205861.3:c.356G > A(p.Arg119Gln)	Missense SNV	*DHDDS*	Putative de novo	VUS (PM2, PP2, BP1); AM = VUS	VUS	M
NED047_P	NM_006734.4:c.5807C > T(p.Thr1936Ile)	Missense SNV	*HIVEP2*	Putative de novo	VUS (PM2, PP2); AM = LP	-	F
**NED082_P**	**NM_014516.4:c.1069G** > **A(p.Ala357Thr)**	**Missense SNV**	***CNOT3***	**Putative de novo**	**VUS (PM2, PP2, BP4); AM** = **LB**	**-**	**M**
**NED082_P**	**NM_005639.3:c.1228G** > **A(p.Val410Ile)**	**Missense SNV**	***SYT1***	**Inherited (M)**	**VUS (PM2, PP2, BP4); AM** = **LB**	**-**	**M**
NED094_P	NM_001348716.2:c.3431G > A(p.Arg1144His)	Missense SNV	*KDM6B*	Inherited (M)	VUS (PM2); AM = VUS	VUS	M
Suggestive							
NED039_P	NM_002860.4:c.2054T > C(p.Ile685Thr)	Missense SNV	*ALDH18A1*	de novo	VUS (PM2, PP3); AM = LB	VUS	M
**NED015_P**	**NM_001370165.1:c.1655A** > **C(p.Lys552Thr)**	**Missense SNV**	***SYTL4***	**X-linked**	**VUS (PM2, PP3); AM** = **LP**	**-**	**M**
NED020_P1 NED020_P2	NC_000019.10:g.49288833_49294972del	Deletion	*SLC6A16*	Inherited (M)	VUS (0)	-	M

SNV/MNV/indels highlighted in bold are unannotated in public databases (GnomAD and ClinVar). Classification of variants according to the ACMG guidelines was performed with Franklin, except in the case of indels, where VarSome was used (*P* pathogenic, *LP* likely pathogenic, *VUS* variant of unknown significance, *LB* likely benign); pathogenicity score for CNVs was calculated with Franklin (<−0.99 = benign, <−0.9 = likely benign, >0.9 < 0.9 = VUS, >0.9 = likely pathogenic, >0.99 = pathogenic); *fs* frameshift, *no-fs* non-frameshift. *pH* pHaplo score, *pT* pTriplo score, *AM* Alpha Missense prediction, *S* SpliceAI score.

*SNV* single nucleotide variant, *MNV* multi nucleotide variant.

**Table 3:** Candidate and suggestive variants in probands affected by ID

Proband		Variant type	Candiate genes	Segregation	ACMG class	ClinVar	Sex
Candidate							
** NED003_P**	**NM_031263.4:c.1326_1343del(p.Asp442_Gln448delinsGlu)**	**Indel (del)**	***HNRNPK***	**de novo**	**LP (PM1, PM2, PM4)**	**-**	**M**
** NED028_P**	**NM_001830.4:c.995T** > **C(p.Leu332Pro)**	**Missense SNV**	***CLCN4***	**de novo**	**LP (PP2, PP3, PM2, PS2); AM** = **LP**	**-**	**M**
** NED038_P**	**NM_182641.4:c.2597_2600del(p.Lys866ArgfsTer16)**	**Indel (del, fs)**	***BPTF***	**de novo**	**P (PVS1, PS2, PM2)**	**-**	**M**
** NED044_P**	**NM_018896.5:c.1174G** > **A(p.Val392Met)**	**Missense SNV**	***CACNA1G***	**de novo**	**VUS (PM2, PP2, PP3); AM** = **LP**	**-**	**M**
** NED088_P**	**NM_002971.6:c.1084G** > **T(p.Glu362Ter)**	**Nonsense SNV**	***SATB1***	**de novo**	**P (PVS1, PS2, PM2)**	**-**	**M**
NED073_P	NM_013275.6:c.1381_1384del(p.Glu461GlnfsTer48)	Indel (del, fs)	*ANKRD11*	de novo	P (PVS1, PP5, PM2)	LP/P	F
NED025_P	NC_000017.11:g.16751634_20572953dup	Duplication	*RAI1* (pT = 0.38)	de novo	P (1)	-	M
NED083_P	NC_000001.11:g.146169841_148577763del	Deletion	*CHD1L, BLC9, GJA8* (pH = 0.04, -, 0.2)	de novo	P (1)	-	M
NED083_P	NM_000531.6:c.264A > T(p.Lys88Asn)	Missense SNV	*OTC*	X-linked	LP (PM1, PM2, PP2, PS1, PP3); AM = LP	LP/P	M
** NED026_P**	**NM_017934.7:c.487C** > **T(p.Arg163Ter)**	**Nonsense SNV**	***PHIP***	**Inherited (M)**	**P (PM2, PVS1, PS4)**	**-**	**M**
NED034_P	NC_000015.10:g.30581193_32433579del	Deletion	FAN1, KLF13, CHRNA7 (pH = 0.5, 0.24, 0.26)	Inherited (M)	P (1)	-	M
** NED036_P**	**NM_153252.5:c.3601A** > **G(p.Arg1201Gly)**	**Missense SNV**	***BRWD3***	**X-linked**	**LP (PM2, PP2, PP3, PM6); AM** = **LP**	**-**	**M**
** NED081_P**	**NM_001160372.4:c.1439_1440del(p.Ser480CysfsTer71)**	**Indel (del, fs)**	***TRAPPC9***	**Inherited (F)**	**LP (PVS1, PM2)**	**-**	**M**
** NED081_P**	**NM_001160372.4:c.1135-8243_1768+2574del(p.?)**	**Deletion**	***TRAPPC9***	**Inherited (M)**	**LP (0.9)**	**-**	**M**
NED073_P	NC_000015.10:g.20019885_23284457dup	Duplication	*NIPA1, NIPA2, TUBGCP5, CYFIP1* (pT = 0.5, 0.4, 0.2, 0.84)	Inherited (F)	VUS (−0.21)	-	F
NED052_P1	NM_001330078.2:c.253C > T(p.Arg85Cys)	Missense SNV	*NRXN1*	Inherited (M/P)	VUS (BP1,PM2); AM = LB	VUS	M
** NED052_P1**	**NM_153252.5:c.4945A** > **G(p.Arg1649Gly)**	**Missense SNV**	***BRWD3***	**X-linked**	**VUS (PM2); AM** = **LB**	**-**	**M**
NED003_P	NM_001008537.3:c.2720A > C(p.Glu907Ala)	Missense SNV	*NEXMIF*	X-linked	VUS (PM2,BP4); AM = LB	VUS	M
Suggestive							
NED003_P	NM_017514.5:c.4703C > G(p.Thr1568Arg)	Missense SNV	*PLXNA3*	X-linked	VUS (PM2); AM = LB	-	M

SNV/MNV/indels highlighted in bold are unannotated in public databases (GnomAD and ClinVar). Classification of variants according to the ACMG guidelines was performed with Franklin, except in the case of indels, where VarSome was used (*P* pathogenic, *LP* likely pathogenic, *VUS* variant of unknown significance, *LB* likely benign); pathogenicity score for CNVs was calculated with Franklin (<−0.99 = benign, <−0.9 = likely benign, >0.9 < 0.9 = VUS, >0.9 = likely pathogenic, >0.99 = pathogenic); *fs* frameshift, *no-fs* non-frameshift. *pH* pHaplo score, *pT* pTriplo score, *AM* Alpha Missense prediction, *S* SpliceAI score.

*SNV* single nucleotide variant, *MNV* multi nucleotide variant.

**Table 4:** Candidate and suggestive variants in probands affected by ASD-ID

Proband		Variant type	Candidate gene	Segregation	ACMG class	ClinVar	Sex
Candidate							
NED005_P	NM_001134407.3:c.1123-1G > A(p.?)	Splicing SNV	*GRIN2A*	de novo	P (PVS1, PP5, PM2); S = 0.99 (splice acceptor)	P	M
** NED063_P**	**NM_001348323.3:c.2359_2360insA(p.Pro787HisfsTer9)**	**Indel (ins)**	***TRIP12***	**de novo**	**P (PVS1, PS2, PM2)**	**-**	**M**
** NED031_P**	**NM_001348323.3:c.1483C** > **T(p.Gln495Ter)**	**Nonsense SNV**	***TRIP12***	**de novo**	**P (PVS1, PS2, PM2)**	**-**	**M**
NED078_P	NM_001374828.1:c.625C > T(p.Gln209Ter)	Nonsense SNV	*ARID1B*	de novo	P (PVS1, PS2, PM2)	-	M
** NED018_P**	**NM_001190274.2:c.2681C** > **T(p.Thr894Ile)**	**Missense SNV**	***FBXO11***	**de novo**	**LP (PS2, PM2, PM6, PP2, PP3); AM** = **LP**	**-**	**M**
NED101_P	NM_005629.4:c.1006_1008del(p.Asn336del)	Indel (del)	*SLC6A8*	de novo	P (PS2, PP5, PP3, PM2)	P	M
** NED077_P**	**NM_014225.6:c.1375C** > **T(p.Arg459Cys)**	**Missense SNV**	***PPP2R1A***	**de novo**	**LP (PS2, PM2, PP2); AM** = **LP**	**-**	**M**
NED001_P	NC_000022.11:g.18751100_21330400del	Deletion	*TBX1, COMT, DGCR8, HIRA, CRKL* (pH = 0.8;0.27;0.8;0.9;0.7)	de novo	P (1)	-	F
** NED080_P**	**NM_013450.4:c.349C** > **T(p.Arg117Ter)**	**Nonsense SNV**	***BAZ2B***	**Inherited (M)**	**LP (PVS1, PM2)**	**-**	**M**
NED067_P	NM_001848.3:c.1138G > A(p.Gly380Arg)	Missense SNV	*COL6A1*	de novo	LP (PM2, PS2, PP3, PP5); AM = LP	LP	M
NED029_P	NC_000007.14:g.152335080_153117170dup	Duplication	*KMT2C*	Inherited (M)	VUS (0.3)	-	M
NED075_P1	NC_000015.10:g.20000921_23357453dup	Duplication	*NIPA1, NIPA2, TUBGCP5, CYFIP1* (pT = 0.53;0.4;0.39;0.88)	Inherited (M)	VUS (-0.62)	-	M
NED080_P	NM_004380.3:c.2558T > G(p.Leu853Arg)	Missense SNV	*CREBBP*	Inherited (M)	VUS (PM2, PP2); AM = LB	-	M
** NED080_P**	**NM_018896.5:c.205C** > **T(p.Arg69Cys)**	**Missense SNV**	***CACNA1G***	**Inherited (M)**	**VUS (PM2, PP2); AM** = **VUS**	**-**	**M**
NED080_P	NM_006245.4:c.1735G > A(p.Val579Met)	Missense SNV	*PPP2R5D*	Inherited (M)	VUS (PM2, PP2, BP4); AM = LP	-	M
**NED037_P1**	**NM_004187.5:c.3561C** > **G(p.Ile1187Met)**	**Missense SNV**	***KDM5C***	**X-linked**	**VUS (PM2, PM6, PP2); AM** = **LB**	**-**	**M**
NED018_P	NM_001543.5:c.2284 G > A(p.Glu762Lys)	Missense SNV	*NDST1*	Inherited (M)	VUS (PM2, PM6); AM = LB	-	M
NED018_P	NM_001543.5:c.2558 G > A(p.Arg853Gln)	Missense SNV	*NDST1*	Inherited (F)	VUS (PM2); AM = LB	VUS	M
NED067_P	NM_020987.5:c.10042A > G(p.Lys3348Glu)	Missense SNV	*ANK3*	Inherited (F)	VUS (PM2, BP4); AM = LB	VUS	F
NED067_P	NM_020987.5:c.11090C > G(p.Ser3697Cys)	Missense SNV	*ANK3*	Inherited (M)	VUS (PM2, BP4); AM = LB	VUS	F
** NED023_P**	**NM_004615.4:c.149T** > **C(p.Leu50Pro)**	**Missense SNV**	***TSPAN7***	**X-linked**	**VUS warm (PM2, PP3); AM** = **LP**	**-**	**M**
** NED040_P**	**NM_001256789.3:c.3609G** > **C(p.Gln1203His)**	**Missense SNV**	***CACNA1F***	**X-linked**	**VUS (PM2, PP3); AM** = **LP**	**-**	**M**
Suggestive							
**NED007_P2**	**NM_001388419.1:c.5327C** > **T(p.Pro1776Leu)**	**Missense SNV**	***KALRN***	**de novo**	**LP (PM2, PS2, PP3, PP2); AM** = **LP**	**-**	**F**
NED059_P	NM_002533.4:c.2182G > T(p.Asp728Tyr)	Missense SNV	*NVL*	de novo	LP (PM2, PP3); AM = LP	-	F
NED029_P	NC_000005.10:g.151891111_151989703dup	Duplication	*GLRA1*	Inherited (F)	VUS (0.3)	-	M
NED029_P	NM_002517.4:c.522+1355_688+1210del(p.?)	Duplication	*NPAS1*	Inherited (F)	VUS (0.3)	-	M
NED029_P	NM_015480.3:c.160+1729_160+5410del(p.?)	Deletion	*NECTIN3*	Inherited (F)	VUS (−0.6)	-	M
NED074_P	NC_000022.11:g.21956425_22222810dup	Duplication	*TOP3B*	Inherited (F)	VUS (−0.35)	-	F
NED074_P	NC_000017.11:g.19340574_20369135del	Deletion	*AKAP10*	Inherited (F)	VUS (0.75)	-	F
NED041_P	NC_000002.12:g.130702734_131311349del	Deletion	*ARHGEF4*	Inherited (M)	VUS (0.65)	-	M
NED051a_P	NC_000009.12:g.152201_384463dup	Duplication	*CBWD1, DOCK8* (pT = 0.2; 0.1)	Inherited (F)	VUS (−0.06)	-	M

SNV/MNV/indels highlighted in bold are unannotated in public databases (GnomAD and ClinVar). Classification of variants according to the ACMG guidelines was performed with Franklin, except in the case of indels, where VarSome was used (*P* pathogenic, *LP* likely pathogenic, *VUS* variant of unknown significance, *LB* likely benign); pathogenicity score for CNVs was calculated with Franklin (<−0.99 = benign, <−0.9 = likely benign, >0.9 < 0.9 = VUS, >0.9 = likely pathogenic, >0.99 = pathogenic); *fs* frameshift, *no-fs* non-frameshift. *pH* pHaplo score, *pT* pTriplo score, *AM* Alpha Missense prediction, *S* SpliceAI score.

*SNV* single nucleotide variant, *MNV* multi nucleotide variant.

We performed SVs discovery on WGS data using both CNVkit^38^, designed to detect copy number variants (CNVs), and a custom SV-discovery pipeline adapted from Vialle et al. ^39,40^ (see Methods). This pipeline integrates the results of multiple bioinformatics tools (Manta, Melt, CNVnator, BreakDancer, Lumpy), to identify deletions, duplications, insertions, and mobile element insertions (Fig. 1 and Methods). Using CNVkit, we initially identified 20,664 CNVs (8809 deletions and 11,855 duplications) (Fig. 2c), while our custom SV-discovery pipeline detected 94,082 SVs, including 50,576 deletions, 8105 duplications, 23,515 insertions and 11,886 MEI (Fig. 2d). Following variant calling, we applied frequency filters to retain only rare events (<1% in gnomAD, CCDG, and the 1000 Genomes Project) located within genes relevant to neurodevelopment. After filtering, 241 CNVs (95 deletions and 146 duplications) and 114 SVs (70 deletions, 44 duplications, 43 insertions, and 25 MEIs) were retained for downstream analyses (Supplementary Data 1 and Table S7–S10). We prioritized de novo and hemizygous SVs in genes with high dosage-sensitivity (pHaplo ≥ 0.55 for deletions; pTriplo ≥ 0.68 for duplications) or pLI (Probability of Loss-of-Function Intolerance) > 0.9 for insertions and MEIs. We also evaluated CNVs affecting genes with lower dosage-sensitivity scores. We believe that the inclusion of potentially suggestive variants from genes below the canonical dosage-sensitivity thresholds may provide valuable insights into complex cases, particularly highlighting genes previously understudied in the context of NDD susceptibility. To assess the likelihood of false positives and evaluate the genotypes and segregation patterns, we manually inspected visualizations generated using CNVkit^38^ and SamPlot^41^ (Figure S1a–u). SVs present in more than ten families, and thus relatively frequent, were excluded as potential contributors to the phenotypes. The most relevant SVs are described in the following sections. Furthermore, to investigate the presence of two potentially gene-damaging variants in different alleles of the same recessive gene, we screened all families to identify probands who inherited at least one filtered SV and one filtered short variant on different alleles, each contributed by a different parent. We identified six probands with both a filtered CNV and a filtered short variant affecting the same gene. However, only one of these variant combinations was considered clinically relevant in the context of NDD phenotype, as discussed in the following sections (Table 3).

### Identification of clinically relevant variants in the NDD cohort

Following variant filtering and manual curation, as detailed in the Methods section, we identified 74 variants of potential relevance in 53 probands (Supplementary Data 1 and Table S11). Among these, 60 were classified as “candidate variants” (47 SNVs/indels and 13 CNVs) located in known NDD-related genes, and 14 were classified as “suggestive variants” (4 SNVs/indels and 9 CNVs) found in genes not yet strongly associated with NDDs but implicated in neurodevelopmental processes based on literature or experimental evidence. Of the 60 candidate variants, 32 (found in 29 probands) were classified as pathogenic/likely pathogenic (P/LP) or warm/hot variants of uncertain significance (VUS), contributing to an overall diagnostic yield of 26.4%.

To further investigate the biological relevance of the affected genes, we examined the 44 unique genes harboring candidate and suggestive SNVs/indels using denovo-db ssc^42^, a curated database compiling de novo variants from large ASD sequencing studies such as the Simons Simplex Collection (SSC). For each variant in our dataset, we assessed three levels of correspondence: (i) *Exact variant match*: whether the same variant was reported as de novo in an ASD proband; (ii) Gene-level match: whether other de novo variants in the same gene were reported in ASD individuals; (iii) Exon-level match: whether de novo variants from ASD cohorts occurred in the same exon, possibly indicating mutational hotspots or shared functional domains (Supplementary Data 1 and Table S12). Among the 44 genes, 33 were present in denovo-db; three of our variants occurred in the same exon as the de novo variants reported in denovo-db ssc, although no exact variant matches were found (Supplementary Data 1 and Table S12). Finally, 52 of the 74 variants (50 candidate and 2 suggestive) affected at least one SFARI gene (Supplementary Data 1 and Table S12).

Then, we further investigated both candidate and suggestive variants within each of the three clinical groups.

### Analysis of genomic variants within the ASD group

We analyzed genomic variants from WGS data of probands in the ASD group (40 cases). These subjects were diagnosed with ASD, did not exhibit clinical phenotypes of ID, and displayed no or less pronounced language impairments compared to those within the ASD-ID group. Importantly, 27 out of 40 probands in this cohort present high-functioning ASD. The genetic architecture of high-functioning autism is complex and may involve both highly and moderately penetrant variants^17,43,44^. For the interpretation of manually curated genomic variants in WGS, we considered that even variants with moderate penetrance, as suggested by in-silico predictions, may be relevant to milder phenotypic presentations. Accordingly, we report several variants with likely moderate penetrance that may contribute to the ASD phenotype (Table 2).

Two rare variants were identified in *SRCAP* (OMIM * 611421) in proband NED016_P, who presented with mild cognitive deficits, behavioral disorder, modest phonological disorder, language and intersubjectivity difficulties, and selective eating habits. The family also reports that the older sister has epilepsy, migraine, psychiatric disorders, and eating disorders; the younger sister presents with a language disorder. Additionally, the father received a diagnosis of ADHD in adulthood. *SRCAP* encodes the core catalytic component of the multiprotein chromatin-remodeling *SRCAP* complex^45^. One variant is a rare splice-acceptor SNV inherited from the mother (NM_006662.3:c.6729+9dup(p.?)), previously classified in ClinVar as a VUS, but predicted as likely pathogenic by in-silico tools (Franklin and AlphaMissense). The second one is an unannotated *missense* SNV inherited from the father (NM_006662.2:c.2494G > A(p.Val832Ile)), classified as a VUS, but located in a highly conserved region (GERP++_RS = 5.36) and predicted to be likely pathogenic (AlphaMissense). *SRCAP* is associated with the Floating-Harbor syndrome (OMIM **#**136140), a language and behavioral disorder, with an autosomal dominant inheritance, and LoF *SRCAP* variants have been found in ASD^46^. The presence of two different variants in the same gene within the family could potentially suggest variable expressivity among family members.

A particularly noteworthy case involved a de novo 660 kbp microdeletion at 1p36.32 (NC_000001.11:g.2538784_3205123del), affecting *MMEL*, *PRDM16*, and *ACTR2*, in a proband with ASD (NED007_P1, Table 5). While classical deletions within the 1p36.31/1p36.32 region are associated with a spectrum of neurodevelopmental conditions, including developmental delay, macrocephaly, brain anomalies, and dysmorphic features^47^, these outcomes are mostly attributed to the deletion of the gene *NFIA*. However, this CNV does not overlap *NFIA*, and it occurs in a proband with high-functioning ASD and without any of the distinctive clinical features of the 1p36.31/1p36.32 deletion syndrome^48^. This microdeletion has also been recently found by Bacchelli et al. and overlaps *PRDM16*, whose Loss-of-function variants have mostly been associated with cardiomyopathy (OMIM#615373)^49^. However, several recent works suggest a crucial role of *PRDM16* in neurodevelopment^50–54^.

**Table 5:** Number of probands affected by potentially pathogenic (P/LP/hot-warm VUS) candidate variants identified in the three experimental groups

Group	Positive	Negative	Total analysed	Yield
ASD	6	34	40	15%
ID	12	15	27	44.4%
ASD-ID	11	32	43	25.6%
**Total probands**	29	81	110	26.4%

A 275 Kbp microdeletion at 16q24.3 (NC_000016.10:g.89640587_89914558del) was inherited from the mother in the family NED082 and overlaps several genes, including dosage-sensitive *CDK10* (OMIM *603464), *CHMP1A* (OMIM *164010), and *FANCA* (OMIM *607139). 16q24.3 microdeletion syndrome is a recently identified disorder associated with variable developmental delay, distinctive facial features, seizures, and ASD. However, the clinical features of 16q24.3 microdeletion syndrome are primarily attributed to haploinsufficiency of the *ANKRD11* (OMIM *611192) gene, which is not involved in the deletion identified in the proband. Nevertheless, de novo variants in the *CHMP1A* gene have been associated with autism^32^. Furthermore, in silico pathogenicity prediction based on ACMG criteria classifies the variant as pathogenic. Notably, the mother, although lacking a formal ASD diagnosis, exhibits ASD-like behavioural traits. It would therefore be advisable to investigate the mother’s phenotype to determine whether the identified variant could be pathogenic but characterized by incomplete penetrance or variable expressivity, a plausible scenario considering that the proband presents with a high-functioning form of autism, in which genetic contributions may sometimes be subclinical or manifest to a lesser extent in carriers.

We also detected a 6 Kbp deletion affecting the last five C-terminus exons of *SLC6A16* (NC_000019.10:g.49288833_49294972del) (OMIM *607972), which was found in both probands and their mother in family NED020. All three individuals share a similar ASD phenotype. While *SLC6A16* is not well characterized, it is expressed in the choroid plexus and may play a role in neuronal function^45^, consistent with its predicted neurotransmitter symporter activities (Supplementary Data 1 and Table S13).

Lastly, we identified two noteworthy single-nucleotide variants of uncertain significance (VUS) in genes not previously implicated in ASD. The first is a de novo VUS in *ALDH18A1* (NM_002860.4:c.2054T > C(p.Ile685Thr)) (OMIM *138250) detected in proband NED039_P. Although this gene, a member of the aldehyde dehydrogenase family, has not been directly associated with ASD, alterations in aldehyde metabolism and toxicity have been implicated in ASD^55^. The second variant is a VUS in *SYTL4* (NM_001370165.1:c.1655A > C(p.Lys552Thr)), which encodes a synaptotagmin-like protein that interacts with Rab GTPases and plays a role in intracellular membrane trafficking^45^. Notably, a likely deleterious missense variant in *SYTL4* was recently reported in an individual with ASD^56^.

In summary, our WGS analysis of 40 probands within the ASD group identified seven pathogenic variants in six probands and sixteen variants of uncertain significance in fifteen probands (Table 2). Among the latter, three occurred in genes not yet strongly associated with ASD. Notably, seven of the detected variants would likely have been missed by exome sequencing, including two CNVs that were not detected by CGH-array analysis. Importantly, 15% (6/40) of the probands in this ASD cohort displayed a disease-causing variant.

These findings highlight a diverse landscape of genetic variation, often of moderate or uncertain penetrance, that may underlie ASD phenotypes in the absence of intellectual disability.

### Analysis of genomic variants within the ID group

We aimed to detect both high-penetrance variants in known ID-causing genes and variants within less characterized genes, which could influence the severity or specific characteristics of the observed phenotypes. Ten out of fourteen pathogenic or likely pathogenic variants were either de novo or X-linked (hemizygous in a male proband) (Table 3).

The remaining four variants were inherited. For instance, in proband NED026_P, we identified a pathogenic nonsense variant in *PHIP* (NM_017934.7:c.487C > T(p.Arg163Ter)) (OMIM * *612870*) inherited from the mother. *PHIP* haploinsufficiency is known to cause a syndrome characterized by behavioral disturbances, intellectual disability, obesity or overweight, and dysmorphic features (OMIM#617991). Consistently, this proband presented with a Sotos-like phenotype, including mild ID, ADHD, and oppositional defiant disorder (ODD), mirroring the clinical features observed in his mother (Supplementary Data 1 and Table S1).

In proband NED034_P, we found a 1.8 Mbp 15q13.2-q13.3 deletion inherited from the mother (NC_000015.10:g.30581193_32433579del) (Table 3). Heterozygous 15q13.3 microdeletions are associated with a wide range of neurodevelopmental conditions, with approximately 75% of these deletions inherited in an autosomal dominant manner. Notably, both the proband’s mother and sister (not enrolled in the study) exhibit a pathological EEG, which could suggest that this CNV contributes to the proband’s phenotype.

In proband NED081_P, we found a combination of two likely pathogenic variants in the *TRAPPC9* (OMIM*611966) gene, one inherited from each parent (NM_001160372.4:c.1439_1440del(p.Ser480CysfsTer71), NM_001160372.4:c.1135-8243_1768+2574del(p.?)). Biallelic deleterious variants in *TRAPPC9* cause a recently described condition associated with non-syndromic intellectual disability and microcephaly (OMIM#613192)^35^ observed only in a limited number of cases to date (*n* = 48 as of 2020), and our case contributes additional allelic and phenotypic information^57^. Notably, one variant is a CNV and the other is an indel, making whole genome sequencing the only approach capable of simultaneously identifying both variants within a single analysis.

Overall, upon manual evaluation of the filtered short variants and SVs, we identified eighteen “candidate” and one “suggestive” variants in thirteen out of twenty-seven probands affected by ID (Table 3). Importantly, WGS allowed us to identify disease-causing variants (deleterious “candidate variants”) in approximately 44.4% (12/27) of the probands in this ID cohort.

### Analysis of genomic variants within the ASD-ID group

The cohort included 43 probands with mid- to low-functioning ASD, typically presenting a complex phenotype involving ID or language impairment (Supplementary Data 1 and Table S1). Among these, several cases illustrated the contribution of inherited variation to complex phenotypes, highlighting the importance of family-based analyses.

**Fig. 3:**
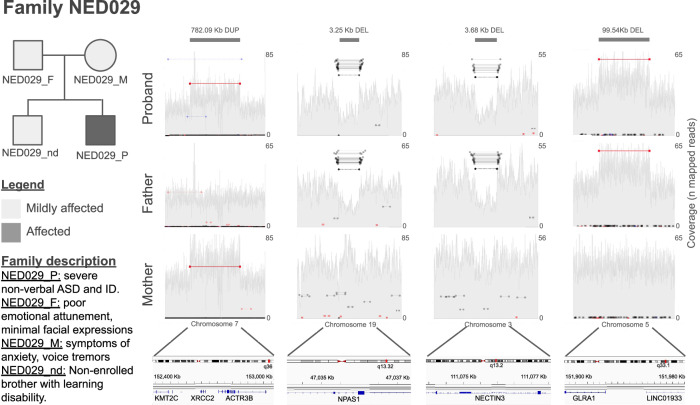
Family structure and CNVs identified in the members of family NED029. Created with Samplot and Microsoft Powerpoint.

Family NED029 exemplifies the value of trio-based WGS. The proband, diagnosed with ASD and ID (Supplementary Data 1 and Table S1), inherited multiple CNVs from both parents (each exhibiting NDD-like traits):: (i) a 780 kbp maternally inherited duplication overlapping the first two exons of *KMT2C* (OMIM*606833) (NC_000007.14:g.152335080_153117170dup) and the whole gene of *XRCC2* (OMIM*600375) and *ACTR3B*; (ii) a 100 kbp paternally inherited duplication of exons 1 and 2 of *GLRA1* (OMIM*138491) (NC_000005.10:g.151891111_151989703dup); (iii) a 3 Kbp paternal deletion overlapping the exon 5 of *NPAS1* (OMIM*603346) (NM_002517.4:c.522+1355_688+1210del(p.?)); and (iv) a 3.7 kbp paternal deletion affecting exon 2 of the transcript ENST00000481766.1 of *NECTIN3* (OMIM*607147) (NM_015480.3:c.160+1729_160+5410del(p.?)). Although neither parent nor the proband’s brother (who was not enrolled in the study) has a formal NDD diagnosis, all present notable behavioural traits. Loss-Of-Function (LoF) variants in *KMT2C* have been associated with autosomal dominant Kleefestra syndrome type-2 (OMIM#617768)^58^, and a partial copy-number gain of this gene has been reported in a child with ASD, poor growth, speech, and developmental delay^59^. *GLRA1* encodes a glycine receptor subunit involved in inhibitory neurotransmission; deleterious variants cause hyperekplexia^60^; and mutant mice exhibit exaggerated startle responses^61^ (Supplementary Data 1 and Table S13). *NPAS1* encodes for a transcription factor with a potential role in neurodevelopment^62^ (Supplementary Data 1 and Table S13), and *NECTIN3* functions in neuronal adhesion, with knockout models showing altered behavior^63^ (Supplementary Data 1 and Table S13). This complex variant landscape may explain the proband’s phenotype and offers insights into the genetic liability in the parents. (Fig. 3).

In NED080_P, the mother shares ASD traits, ID, and severe obesity with the proband. We identified a maternally inherited likely pathogenic variant in *BAZ2B* (OMIM*605683) gene (NM_013450.4:c.349C > T(p.Arg117Ter)), a recently proposed ASD/ID gene^64^, which is also downregulated in adipose tissue of individuals with obesity^65^.

We also identified a likely pathogenic de novo SNV in *COL6A1* (OMIM*120220) (NM_001848.3:c.1138G > A(p.Gly380Arg)) in proband NED067_P. Although this gene has not been previously implicated in ASD, *COL6A1* knockout mice display marked behavioral abnormalities^66^. Furthermore, the motor impairment component characterizing the probands phenotype is consistent with disorders associated with this gene, including Bethlem myopathy (OMIM#158810) and Ullrich congenital muscular dystrophy (OMIM#254090). In addition, we detected two VUS in *KALRN* (NM_001388419.1:c.5327C > T(p.Pro1776Leu)) and *NVL* (NM_002533.4:c.2182G > T(p.Asp728Tyr)), both of which are involved in neurodevelopmental regulation^67^^,^^68^ (Supplementary Data 1 and Table S13).

Overall, in the ASD-ID group, we identified 22 “candidate” causative variants in eleven probands (approximately 25.6%, 11/43) and nine “suggestive”" variants in six probands (Table 4).

### WGS reveals a higher diagnostic yield in the ID group

To evaluate whether the three diagnostic groups differed in their predicted diagnostic yield, we performed a chi-squared test, which revealed a statistically significant difference between groups (*χ*² = 6.002, df = 2, *p* = 0.049), suggesting that the likelihood of identifying deleterious ‘candidate’ variants is not evenly distributed across clinical subtypes. Notably, individuals with isolated ID showed a higher diagnostic rate compared to those with ASD or combined ASD-ID phenotypes.

We also performed pairwise comparisons between groups using Fisher’s exact test. The difference between the ID and ASD-ID groups did not reach statistical significance (*p* = 0.1223), although there was a trend toward a higher diagnostic rate in the ID group. This lack of statistical significance may be attributable to the limited cohort size. No significant difference was found between the ASD and ASD-ID groups (*p* = 0.4318). In contrast, individuals with ID showed a significantly higher diagnostic yield compared to those with ASD-only (*p* = 0.0263).

These results suggest that individuals with isolated ID may have a higher likelihood of receiving a molecular diagnosis through whole genome sequencing than those with ASD or combined phenotypes ASD-ID, although the limited sample size warrants cautious interpretation.

### Evo 2 analysis highlights potentially deleterious VUS variants

We applied the Evo 2^69^ algorithm to score all short variants (SNVs and Indels) described in the previous sections (Tables 2–4; Supplementary Data 1 and Table S14). In parallel, we analyzed 17,054 ClinVar variants to compare the distribution of Evo 2 scores for our variants with those of variants having well-defined clinical interpretations in the same genes (Supplementary Data 1 and Table S15; Supplementary File 1 and Fig. S1).

**Fig. 4:**
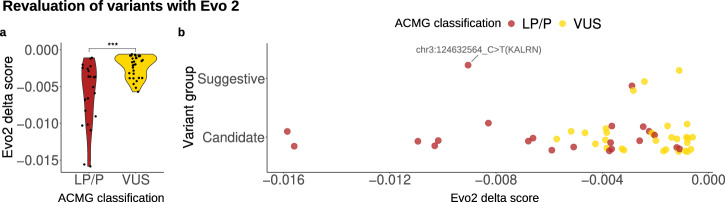
Re-evaluation of variants using Evo 2. **a** Distribution of Evo 2 delta scores for variants previously classified as pathogenic/likely pathogenic (P/LP) or of uncertain significance (VUS) according to ACMG guidelines (***p-value < 0.001); **b** Distribution of Evo 2 delta scores for variants classified in this study as candidate or suggestive. Created with R (ggplot2 package).

Pathogenic or likely pathogenic (P/LP) variants in NDD probands showed significantly lower Evo 2 delta scores (*p*-value < 0.001) than VUS (Fig. 4a), supporting the potential of Evo 2 delta scores as predictors of variant deleteriousness in NDDs.

Interestingly, eight candidate VUS displayed Evo 2 delta scores comparable to those of ClinVar entries annotated as P/LP in the same genes (Supplementary Data 1 and Tables S14 and S15; Supplementary File 1 and Fig. S1). These include: NM_004380.3:c.2558T > G (p.Leu853Arg) in *CREBBP* (proband NED080_P), NM_001368397.1:c.3598T > C (p.Ser1200Pro) in *FRMPD4* (proband NED020_P2), NM_006734.4:c.5807C > T (p.Thr1936Ile) in *HIVEP2* (proband NED047_P), NM_004187.5:c.3506C > G (p.Ser1169Trp) in *KDM5C* (proband NED055_P), NM_000240.4:c.608G > A (p.Gly203Asp) in *MAOA* (proband NED049_P1), NM_001008537.3:c.2720A > C (p.Glu907Ala) in *NEXMIF* (proband NED003), NM_001374828.1:c.588_589insAGCAGCAGCAGCAGCAGCAGCAAC (p.Phe196_Gln197insSerSerSerSerSerSerSerAsn) in *ARID1B* (proband NED082_P), and NM_152641.4:c.3197C > T (p.Pro1066Leu) in *ARID2* (proband NED092_P). In particular, variants within *HIVEP2*, *MAOA*, and *ARID2* were also classified as LP by the AlphaMissense algorithm, suggesting that they may warrant re-evaluation as ACMG criterion and be considered as C4 candidates, potentially contributing to an increased diagnostic yield.

Furthermore, all five suggestive SNV/indels described (Tables 2–4) showed lower delta scores than most ClinVar VUS in the corresponding genes (Supplementary Data 1 and Table S14), supporting their potential functional relevance. Among these, the de novo variant in *KALRN* NM_001388419.1:c.5327C > T(p.Pro1776Leu) (proband NED007_P2) is particularly noteworthy. This variant not only displays a delta score lower than all ClinVar VUS in *KALRN* but also lower than most P/LP variants described in our study (Fig. 4b), and it is classified as LP by Alphamissense. The variant was identified in a proband with ASD with severe motor impairment and difficulties in handling objects, mild ID, language delay, motor and verbal stereotypies, mild selective eating, and epileptic episodes. The *KALRN* gene (OMIM *604605) encodes a multidomain guanine nucleotide exchange factor (GEF) for Rho family GTP-binding proteins and has been proposed to play a crucial role in synaptic regulation^68,70,71^. Homozygous variants in *KALRN* have been reported as causative of intellectual disability^72^, while alterations in *KALRN* have also been implicated in schizophrenia^73^ and epilepsy^74^ in experimental models. The extent to which this gene influences NDD phenotypes, and whether it does so via a dominant or recessive inheritance pattern, remains a matter of debate. Nevertheless, taken together, these observations provide strong support for *KALRN* as a promising novel candidate gene for ASD.

### Secondary findings

Whole genome sequencing enables the detection of pathogenic variants not directly associated with NDDs but falling within the list of 84 genes recommended for clinical reporting by the American Society of Human Genetics^75^. Variants in these genes are associated with diseases for which preventive measures, surveillance, or treatments are available, thus representing actionable findings that may significantly improve health outcomes. These variants are commonly referred to as “*secondary findings*”. In our cohort, we identified three clinically relevant secondary findings: (i). A pathogenic hemizygous variant in *OTC* gene (NM_000531.6:c.264 A), in proband NED083_P, inherited from the mother. This variant is associated with ornithine transcarbamylase deficiency (OTCD, OMIM#311250), an X-linked recessive metabolic disorder that can lead to hyperammonemia and neurological complications. As previously discussed, the proband also exhibits ID and carries a likely pathogenic CNV at 1q21.1, suggesting a complex genetic background.; (ii). a heterozygous pathogenic variant in *MYBPC3* (NM_000256.3:c.2459G > A), identified in the mother of NED093_P. This variant is associated with hypertrophic cardiomyopathy, an autosomal dominant cardiac condition characterized by variable expressivity and risk of sudden cardiac death^35^; (iii). a heterozygous pathogenic variant in *BRCA2* (NM_000059.4:c.9117G > A) found in the mother of NED054_P. *BRCA2* deleterious variants confer an increased risk for hereditary breast and ovarian cancer syndromes, following an autosomal dominant inheritance^35^. This finding has important implications for cancer screening and familial risk assessment.

These cases exemplify the potential of WGS to uncover medically actionable information beyond the primary diagnostic intent, underscoring the utility of genomic approaches in precision medicine.

### Independent validation of relevant variants

We aimed to validate the most relevant genomic variants identified through WGS using independent methods in order to exclude potential artefacts arising from alignment or variant calling errors.

Of the 55 SNVs and indels identified through WGS and classified in our study as candidate, suggestive or secondary findings, we were not able to design appropriate primers in 4 cases, primarily due to the presence of repetitive elements, high GC content, or other sequence complexities at the *locus* that interfered with efficient primer binding or PCR amplification (Supplementary Data 1 and Table S16). Overall, the validation rate was 100%, supporting the reliability of NGS-based variant detection and consistent with reported validation rates in literature (91.29%–100%, depending on variant calling quality)^76^.

To assess the reliability of our CNV calls, we conducted a visual validation of all 21 CNVs classified as candidate or suggestive by manually inspecting read-depth plots generated with CNVkit and Samplot, which enable the visualization of sequencing coverage across the genomic regions encompassing the predicted CNVs (Supplementary File 1 and Fig. S2a–u). Among these 21 CNVs, 13 were successfully validated using independent methods (Supplementary Data 1 and Table S16). Specifically, nine had previously been identified through array Comparative Genomic Hybridization (CGH-array), and our analysis of WGS data confirmed their presence in the probands and enabled us to assess their segregation in the parents (Supplementary File 1 and Fig. S2a–u). Four CNVs were successfully validated using a PCR-based method specifically designed for the corresponding *loci*. The remaining eight CNVs could not be validated through the available approaches. In two cases, PCR validation was attempted but failed due to non-functional primers, likely because of the high content of repetitive sequences in the targeted regions. Five CNVs are currently scheduled for PCR validation and remain unconfirmed. Another CNV could not be assessed via CGH-array because the analysis was not performed on the proband. Given that the array platform employed probes spaced 40–60 Kbp apart, we hypothesize that CNVs smaller than approximately 200 Kbp may fall below the resolution of the assay. Nevertheless, four CNVs, although relatively large in size (greater than 230 Kbp), were not reported in the CGH-array results. Nonetheless, the absence of these specific CNVs in the CGH-array data is difficult to fully interpret, particularly because visual inspection of the sequencing read-depth plots provides clear evidence supporting their presence (Supplementary File 1 and Fig. S2a–u). Overall, these data indicate that whole genome sequencing may be more accurate than CGH-array for the identification of CNVs, although further validation is needed to confirm this across different CNV lengths.

## Discussion

We present a comprehensive WGS analysis to investigate rare genomic variants that contribute to NDDs, with a focus on ASD and ID. Our study cohort consisted of 100 families, including 110 probands affected by NDDs.

The probands exhibited a broad range of NDD phenotypes, from severe ASD with profound cognitive impairment to high-functioning ASD with mild presentations. The inclusion of individuals with milder phenotypes is particularly valuable, as these cases are often underdiagnosed and under-represented in genomic studies. Analyzing these cases may help clarify the genetic contribution to these forms of ASD. Although most families included two healthy parents, 18 families had at least one parent with NDD-like traits, which was considered in the interpretation of segregation and variant effects.

Through an integrative approach, we identified damaging de novo and inherited variants across different classes, including SNVs, indels, SVs, INSs, and MEI, which helped expand the allelic spectrum and elucidate diverse inheritance patterns across NDD-associated and candidate genes. While no INS or MEI of clinical significance was observed, multiple SNVs, indels, and SVs emerged as promising candidates for further analysis.

In total, we identified 32 potentially phenotype-causing variants in 27 out of 110 probands (26.4%), comprising 23 short variants and 9 SVs. Additionally, we report 41 variants of uncertain significance (VUS) we deemed worthy of further investigation (29 short variants, 12 SVs). No relevant variants were detected in approximately 52% of the probands (57/110).

We identified 24 likely pathogenic single-gene variants in 20 genes previously associated to NDDs, including *TRAPPC9*, *HNRNPK, CLCN4, BPTF, CACNA1G, SATB1, ANKRD11, OTC, PHIP, BRWD3, GRIN2A, TRIP12, ARID1B, PPP2R1A, FBXO11, SLC6A8, TSPAN7, KMT5B, ATP1A3, BAZ2B, COL6A1*, and *SRCAP*. We also detected three de novo protein-altering SNVs in *KALRN, NVL*, and *ALDH18A1*, genes not currently recognized as high-confidence ASD-ID *loci*. These probands were all affected by ASD with different degrees of severity, suggesting these genes warrant further functional exploration.

Two VUS were identified in *PLXNA3 and SYTL4*, and seven SVs of unknown significance affecting *GLRA1, NPAS1, NECTIN3, TOP3B, AKAP10, SLC6A16*, and *ARHGEF4*, none of which are well established in NDD etiology.

25 VUS were identified in 22 known NDD-causative genes, including *NRXN1, BRWD3, NEXMIF, CREBBP, CACNA1G, PPP2R5D, KDM5C, NDST1, ANK3, CACNA1F, ARID1B, ARID2, KDM5C, FRMPD4, MAOA, CACNA1D, DHDDS, HIVEP2, CNOT3, ANKRD11, SYT1*, and *KDM6B*. In addition, 12 large CNVs were found in 9 recurrent NDD-associated *loci*: 17p11.2, 1q21.1, 15q13.2-q13.3, 22q11.2, 1p36.32, 15q11.2, 9q24.3, 7q36.1/7q36.2, and 16q24.3.

The clinical significance of variants followed ACMG guidelines^77^, resulting in 12 variants classified as likely pathogenic and 17 as pathogenic in known NDD genes (Tables 2–4); although the remaining three variants were classified as VUS by in silico analyses, we nonetheless included them as potential phenotype-modifying candidates following a rigorous critical evaluation. Overall, we estimate a diagnostic rate of 26.4%, consistent with previous WGS-based studies^18,19^. Notably, 11 of the 32 phenotype-causing variants would have been missed by WES due to their genomic localization in non-coding regions, and one likely pathogenic CNV was not detected by CGH-array, supporting an estimated ~10% increase in diagnostic yield when using WGS compared to WES.

Reported diagnostic yields for genome/exome sequencing in NDDs vary widely by cohort composition, ascertainment, and analytic strategy. Meta-analyses estimate yields around 31-36%, with higher rates in consanguineous or deeply phenotyped cohorts. Our lower yield (26.4%) likely reflects the heterogeneity of our cohort of non-consanguineous families, which includes many high-functioning ASD cases with potentially stronger polygenic contributions^44^, and its modest sample size, which increases variability.

In addition, following a detailed re-evaluation of VUS in collaboration with the referring clinicians, taking into account family history and phenotypic data, we anticipate that some of these variants may ultimately be reclassified as likely pathogenic (class 4). Consequently, the reported diagnostic yield may represent a conservative (downward) estimate.

Furthermore, re-evaluation of variants using Evo 2^69^ indicated that eight VUS might be reconsidered as likely pathogenic, potentially leading to an increase, though modest, diagnostic yield. The observation that several VUS displayed Evo 2 delta scores comparable to those of ClinVar P/LP variants in the same genes suggests that evolutionary modeling approaches can refine the prioritization of variants in NDD cohorts. These findings are consistent with recent evidence supporting the use of large-scale language models trained on genomic sequences to capture functional constraints beyond conventional conservation metrics^78^. Moreover, the algorithm contributed supportive evidence for the deleterious effect of a de novo suggestive variant in *KARLN*, highlighting it as a potential candidate gene for NDDs despite the current lack of a well established association. Importantly, Evo 2 is also expected to be highly valuable for evaluating the potential clinical relevance of non-coding variants, an area that remains particularly challenging in genetic diagnostics. While our study focused on coding variants that alter protein function, WGS provides access to the non-coding genome, harboring regulatory elements such as enhancers, silencers, insulators, and transcription factor binding sites. Disruption of such regulatory elements may significantly impact gene expression and contribute to disease pathogenesis^23^. We speculate that non-coding variants may account for a subset of unsolved cases and plan to implement genome-wide regulatory variant annotation. Although Evo 2 has not yet undergone full peer-reviewed, our preliminary results suggest it may meaningfully refine variant interpretation.

Interestingly, we observed a stepwise increase in diagnostic yield, from the lowest in ASD, to intermediate in ASD-ID, and the highest in ID. This aligns with prior studies^79^ and may reflect the more severe and clearly delineated phenotypes typically observed in the ID group, which are more often attributable to rare, highly penetrant variants. In contrast, the ASD-only subgroup includes several high-functioning individuals whose phenotypes are more likely influenced by polygenic and environmental factors^44,80^, reducing the likelihood of identifying a single causative variant. When considering the ASD-ID group, no significant difference was detected relative to ASD-only, suggesting broadly comparable rates of monogenic diagnoses between these two subgroups. At the same time, the absence of statistical significance may simply reflect the limited sample sizes available for subgroup analyses.

**Fig. 5:**
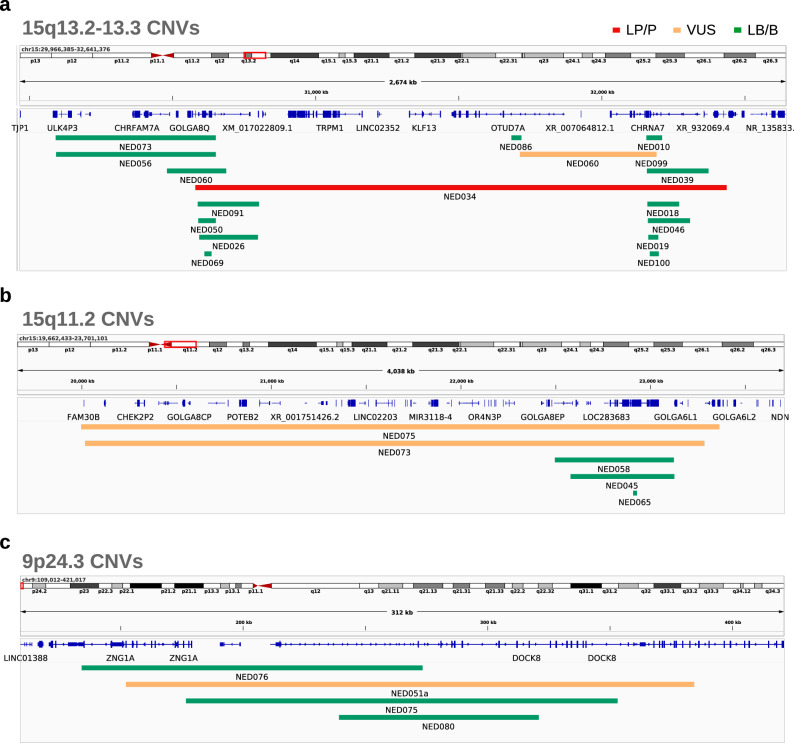
Potentially phenotype-relevant recurrent CNVs identified in multiple analyzed individuals. **a** CNVs found in locus 15q13.2-13.3; **b** CNVs found in locus 15q11.2; **c** CNVs found in locus 9p24.3. B/LB benign/likely benign, VUS variant of unknown significance, LP/P likely pathogenic/pathogenic. Created with IGV (Integrative Genomic Viewer) and LibreOffice Draw.

We also assessed CNVs known for incomplete penetrance and variable expressivity, such as those affecting 15q13.2-q13.3, 15q11.2, and 9p24.3 (Fig. 5a–c and Table 5). Notably, all CNVs mapping into chr15 in probands were inherited from the opposite sex parent (father to female proband or mother to male proband). This is intriguing given that these CNVs are located in the immediate upstream and downstream loci from the canonical imprinted region on chromosome 15q11-13 (Table 6 and Fig. 5b).

**Table 6:** Potentially phenotype-relevant recurrent CNVs identified in multiple analyzed individuals

Locus	Genomic coordinates	Carriers	CNV type
9p24.3	chr9:239418-320947	NED080_F	DUP
9p24.3	chr9:134189-273546	NED076_F	DUP
9p24.3	chr9:176849-356023	NED075_F	DUP
9p24.3	chr9:152201-384463	NED051a_P, NED051a_F	DUP
15q11.2	chr15:22477726-23280665	NED073_P, NED073_F	DUP
15q11.2	chr15:21043650-23357453	NED075_P, NED075_M	DUP
15q11.2	chr15:22571606-23125407	NED045_P, NED045_F	DEL
15q11.2	chr15:22553589-23122562	NED058_F	DEL
15q13.3	chr15:31714997-32155815	NED060_P, NED060_M	DUP
15q13.2-q13.3	chr15:30581192-32233551	NED034_P, NED034_M	DEL

We identified four 9p24.3 duplications partially overlapping *DOCK8*, a gene previously implicated in learning disabilities and behavioral disturbances, with cases of incomplete penetrance^81^^,^^82^ (Table 5 and Fig. 5c). The largest duplication was detected in a male proband with ASD-ID (NED051a_P) and his father, while the other three were detected in fathers not transmitting the variant. One carrier father (NED076_F) reported a learning disability, suggesting incomplete penetrance and phenotypic variability (Supplementary Data 1 and Table S1). While the interpretation of these observations is uncertain, these results may help improve the understanding of the impact of partial *DOCK8* duplications in NDDs.

It is important to note that the results presented in this work represent a subset of data from a larger research initiative, “5000genomi@VdA,” which involves the WGS of approximately 5000 individuals from the Valle d’Aosta region. This broader dataset, which includes a substantial proportion of healthy individuals from the same genetic background as the NDD cohort, provides an opportunity to refine our analyses and interpretation of the variants identified in this study in the future. By integrating the NDD cohort with this larger population reference, we plan to investigate the potential contribution of common coding and noncoding variants that may not be captured by conventional diagnostic pipelines and to enhance the detection of structural variants and complex rearrangements through population-based calibration.

Although not related to the initial clinical indication for this NDD cohort, the large amount of data generated by WGS enabled the identification of a few secondary findings. In this study, we also outline a procedure for their effective communication to study participants, which is relevant for implementing preventive healthcare strategies and treatments.

Collectively, these results support WGS as a superior diagnostic tool compared to WES or other targeted approaches, given its ability to detect diverse and complex variant types. A key limitation of our study is the relatively small cohort size, which restricts power for novel gene discovery. Nevertheless, we believe that the family-based design and the analysis of an under-represented population provide valuable insights into the genetic architecture of NDDs and establish a foundation for future research and validation.

## Methods

### Study cohort

Participants were enrolled in a prospective monocentric observational study, entitled “*Neurodevgenomics*,” designed to investigate the genomic alterations in families with ASD and/or ID resident in Valle d’Aosta. Pediatric and adult patients (age ratio 1-42 years old, male-to-female ratio 2.5:1) (Supplementary Data 1 and Table S1) and their parents were recruited through the SDD Pediatric Neuropsychiatry of Beauregard Hospital and SC Psichiatry of Umberto Parini Regional Hospital, Aosta, Italy, in the period between May 2022 and November 2024. Blood or saliva samples were collected from probands, their parents, and affected siblings, totalling 298 individuals (Supplementary Data 1 and Table S1). The study cohort included 100 families, encompassing 110 probands diagnosed with ASD or ID or both. Probands with severe language impairment were included in the ASD-ID group, as formal assessment of intellectual functioning was not feasible due to the limitations imposed by their language deficits.

The *Neurodevgenomics* study protocol was approved by the Institutional Review Board (IRB) of the Umberto Parini Regional Hospital in Aosta during the review meeting held on March 24, 2022, as documented in the official report (Protocol No. *CE PROTOCOLLO.I.0029523.05-04-2022*). Written informed consent was obtained from all the subjects involved or from their Legally Authorized Representatives, including consent for publication of anonymized genetic and clinical details. Investigations were conducted in accordance with the ethical standards as outlined in the Declaration of Helsinki.

### Clinical assessment and proband stratification

Clinical diagnosis followed DSM-5 criteria^83^ and was conducted by a multidisciplinary team. Evaluations included standardized tools to measure cognitive and developmental levels (Wechsler Scales, Griffiths Scales, PEP-3, Leiter-R) and adaptive behavior (Vineland Adaptive Behavior Scale, VABS; Adaptive Behavior Assessment System, ABAS-2)^84,85^. For suspected ASD cases, diagnostic and severity assessments were conducted using the Autism Diagnostic Observation Schedule, Second Edition (ADOS-2)^86^. IQ scores were categorized into five levels (severe, moderate, mild, borderline, normal) to ensure consistency across various measurement tools.

### Whole genome sequencing

Genomic DNA (gDNA) was extracted from peripheral blood of all participants, except for two individuals NED014_F and NED032_F for whom gDNA was extracted from saliva. Short-read whole genome sequencing (srWGS) of gDNA was conducted on an Illumina NovaSeq 6000 platform (Illumina Inc., CA, USA) to produce paired 151 base-pair reads. The library preparation and sequencing of gDNA utilized barcode adapters. Specifically, 350 ng of gDNA was prepared using the Illumina DNA PCR-Free Prep, Tagmentation library preparation kit (Cat number 20041795), and IDT® for Illumina® DNA/RNA Unique Dual Indexes Set A and B, Tagmentation (96 Indexes, Cat numbers 20027213 and 20027214). The library preparation was carried out with a T100 Thermal Cycler instrument (Bio-Rad) and standard laboratory equipment. Library quantification was performed with KAPA qPCR Library Quantification Kit (Roche, Cat number 0 7960140001). Libraries were pooled by volume, adjusted for the index correction factor according to Illumina’s instructions to ensure balanced sample coverage across each run. An average sequencing depth of approximately 38X was achieved for all samples (Supplementary Data 1 and Table S3–S5).

### Validation of SNVs and SVs

SNVs and Indels were validated with Sanger sequencing. For each variant, DNA amplification was performed under different conditions depending on product length and melting temperatures of the designed primers (see Supplementary Data 1 and Table S16). The Sanger sequencing was performed using the SeqStudio Genetic Analyzer (ThermoFisher Scientific, CA, USA) according to the manufacturer’s specification. Negative controls confirmed the absence of amplification inhibitors. DNA quality and quantity were assessed before genotyping. Primers were designed using Primer-BLAST from NCBI^87^ and synthesized by Sigma-Aldrich (USA). To validate SVs via PCR amplification, forward and reverse primers were designed to flank the breakpoints of deletions or duplications, ensuring specificity by avoiding regions prone to non-specific annealing. Owing to the unique configuration of the variants, PCR products were observed exclusively in carrier individuals. Information for the validated variants and primer sequences is presented in Supplementary Data 1 and Table S16.

CGH-array had been previously performed on blood-derived samples for all probands, except for NED034_P, at Medical Genetics Unit, Molinette Hospital, Turin, Italy. CGH-array analysis was performed using the AGILENT SurePrint G3 60 K platform, which uses genome-wide probes with an average spacing of 60 Kbp. The identification of a deletion or duplication requires a significant deviation from the normal value in at least 3 adjacent probes. The CytoGenomics 5.2 software was used to analyze the data.

### Kinship analysis

We assessed the kinship of all families using Plink^88^ (v1.90b6.21) and KING^89^ (v2.2.6) on quality-filtered short variants detected with HaplotypeCaller (Parabricks). Results of the kinship analysis are available in Supplementary Data 1 and Table S2.

### List of neurodevelopmentally-relevant genes

Evaluating the genomic causes of NDDs is challenging. In addition to well-established NDD genes, many genes are not yet recognized as definitive NDD genes but may still significantly contribute to the neurodevelopmental phenotypes. To address this issue, we compiled a comprehensive list that includes: (i) genes present in the SFARI (Simons Foundation Autism Research Initiative) database (January 2024 release)^32^; (ii) genes with high brain expression, defined as those genes with average log2 RPKM > 4.5 in the BrainSpan database (http://www.brainspan.org) (the top 18%)^90^; (iii) genes of the KEGG pathways^91^ (https://www.genome.jp/kegg) selected for their possible relevance with ASD (including Nervous system, Genetic Information Processing, and Replication and repair); (iv) genes associated to neurodevelopmental phenotypes according to OMIM^35^. However, the role of some genes in NDDs may be supported only by recent evidence. We therefore aimed to ensure that potentially relevant genes were not overlooked in our analysis. To achieve this, we conducted an initial analysis of genomic variants across all annotated human protein-coding genes, with a particular focus on de novo and X-linked variants. All genomic variants described in this work have been found within genes included in this updated list (available in Supplementary Data 1 and Table S17).

### Identification and prioritization of short variants

We used the NVIDIA Clara™ Parabricks® pipeline (version 3.8) with BWA-mem to map raw sequencing reads to the reference genome (hg38). We then performed germline short variant calling (SNVs and indels) with the NVIDIA Clara™ Parabricks® germline pipeline (3.8 version) with GATK HaplotypeCaller^92^. Called variants were filtered in order to retain only variants labeled as “PASS”. Filtered variants were annotated using Annovar (databases updated to 04/01/2024).

We then filtered total variants in order to only retain variants with the following characteristics: (1) present in less than 0.5% of the population (GnomAD v4^93^), in less than 5 times in heterozigosys, and less than 3 times in homozigosys in the internal cohort (composed of 298 individuals); (2) annotated as “exonic” or “splicing”; (3) not synonymous; (4) not annotated as “Benign” or “Likely Benign” by InterVar^94^ or ClinVar^95^; (5) associated with a CADD_phred score^96^ above 20 (or unassigned); (6) present in the proband and supported by at least 10 reads in at least one member of the family, including the proband; (7) identified within a gene included in a curated list of 4264 genes of interest in the context of neurodevelopment (Supplementary Data 1 and Table S17). The application of these filters resulted in ~37 variants per family, which we refer to as ‘filtered variants’. Additionally we assigned one of the following inheritance patterns to each filtered variant: inherited (when present in heterozygosity in a parent and a proband); de novo (variants present only in the proband); recessive (variants present in homozygosity in the proband and in heterozygosity in both parents); X-linked variants (variants present in hemizygosity in male probands and in heterozygosity in the mother); “composite recessive” variants (instances where more than at least one variant per parent is inherited in the same gene in the proband) and *‘unknown’* when we were not able to clearly identify the inheritance pattern. We manually evaluated filtered variants employing the following criteria: (1) relevance of the gene in the context of the probands phenotype (assessed using databases OMIM/ORPHANET and literature review); (2) genes inheritance mode (when specified by OMIM); (3) ACMG guidelines (especially in the case of known disease-causing genes, by using the computational tools InteVar, VarSome and Franklin), moreover, we considered the AlphaMissense score^97^ when evaluating *missense* variants; (4) protein domain overlapping the variant (assessed with UniProt); (5) overall damaging potential of the variant (CADD_phred score); (6) evolutionary conservation of the genomic locus (GERP++_RS score^98^); family structure (especially when one or both parents present a similar phenotype compared to the proband).

We performed manual curation to identify gene-damaging variants within known NDD-causing genes, which we define as candidate variants. We also identified potentially deleterious variants in genes not clearly associated with a specific phenotype but for which evidence suggests involvement in neurodevelopment; these are defined as “suggestive variants”. All missense candidates and suggestive variants were also classified as either LB, VUS, or LP with Alphamissense^97^.

### Identification and prioritization of structural variants

Upon mapping raw sequencing reads to the reference genome (hg38) with NVIDIA Clara™ Parabricks® pipeline (version 3.8), we used two methods to detect structural variants: a custom pipeline comprising multiple SV-caller bioinformatics tools (adapted from Vialle et al. ^39,40^) to identify CNVs (deletions and duplications) smaller than 500 kb, insertions (INS), and mobile element insertions (MEI); and CNVkit (v0.9.9) to detect larger CNVs. The pipeline adapted from Vialle et al. ^39^. uses four tools to call CNVs (Manta^99^ (v1.6.0), CNVnator^100^ (v0.3.3), BreakDancer^101^ (v1.4.5), and Lumpy^102^ (v0.2.13)) and reports only CNVs identified by at least two of these four tools. Additionally, this pipeline calls insertions with Manta^99^ and non-reference mobile element insertions with Melt^103^.

We run CNVkit batch analysis to detect CNVs against a reference genome constructed with 10 genomes from healthy individuals, with “*hmm-germline*” segmentation method and “*wgs*” seq-method. We filtered identified CNVs to discard signals from background noise or non-germline CNVs (log2 copy-number > 0.35 or < −0.4, *p*-value < 10E^-05^, weight > 20). Moreover, we merged the genomic coordinates of CNVs of the same type (deletions or duplications) closer than 50 Kbp to each other.

Both in the case of variants called with CNVkit and with the SV pipeline adapted from Vialle et al., we merged the variants relative to the members of each family using SURVIVOR to generate family-level multi-sample VCF files. These files were then annotated with SVAFotate and AnnotSV (v3.2.3). We selected only rare variants (frequency < 0.01) overlapping exons of neurodevelopmentally-relevant genes (Supplementary Data 1 and Table S17) that were identified in at least one family member. We also filtered out all CNVs present in more than 10% of enrolled families. In the case of INS and MEI, we retained only variants found at less than 20 bp from one exon of a neurodevelopment-related gene. All filtered CNVs were subjected to manual curation. We generated and visually inspected family-level plots generated with the CNVkit scatterplot utility in the case of variants called with CNVkit, and with SamPlot46 in the case of structural variants identified with our custom pipeline. This allowed us to estimate the likelihood of false positives, verify variant inheritance within trios, and identify variants that might not have been formally called in all family members despite being present. Following validation of variant authenticity, the pathogenic potential of CNVs we deemed worthy of further investigation was evaluated according to ACMG guidelines using Franklin (Genoox) and VarSome.

### Evaluation of genomic variants

To identify potentially phenotype-causing variants, we mainly focused on de novo, *X-linked*, and homozygous variants. Analysis of de novo SNVs/SVs variants was performed only for complete trios (89 out of 100 families); we took into account the genotype reported by HaplotypeCaller for short variants, by Melt for MEI, and by Manta for INS. We assessed the genotype of CNVs manually by inspecting plots produced with CNVkit and SamPlot.

However, we manually evaluated inherited variants as well, especially in the cases where one or both parents presented phenotypic traits similar to the ones observed in the proband. We divided variants of potential interest into two main categories: candidate and suggestive variants.

Candidate variants are variants affecting genes that are known to be involved in a neurodevelopmental phenotype that matches the proband’s phenotype, following a specific inheritance pattern observed in the variant. Candidate variants can be classified as either pathogenic (P), likely pathogenic (LP), variants of uncertain significance (VUS), or as VUS with strong supporting evidence (referred to as “warm/hot” VUS) according to the ACMG guidelines. The diagnostic yield in this study was driven exclusively by candidate variants classified as P, LP, or hot/warm VUS. Suggestive variants are those classified as VUS or P/LP according to ACMG guidelines, located in genes not yet strongly associated with NDDs but reported in recent literature as potentially relevant to human neurodevelopment.

### Variant evaluation with Evo 2

To provide additional evidence supporting the variants identified in this study, we applied Evo 2^69^, a recently developed machine learning-based prioritization framework that integrates measures of evolutionary constraint, gene-level intolerance, and variant pathogenicity scores to refine variant classification.

For each short variant described in the previous paragraphs (Tables 2–4), we computed the Delta likelihood scores using the Evo 2 7B model in a zero-shot setting. For each variant, its genomic position was located on the corresponding chromosome, and an 8000 bp window centered around that position was used to extract the variant and reference sequence pairs. For SNVs, the variant nucleotide replaced the reference nucleotide at the center of the variant sequence. For deletions, the deleted segment was removed after the given position, and the 8000 bp window was extended into the neighboring regions to maintain the total sequence length (8000 bp). For insertions, the inserted nucleotides were added after the specified position, and an equivalent number of nucleotides were removed from the end of the variant sequence to keep the length consistent. In addition to the original variant and reference sequences, their reverse complements were also used to calculate log-likelihoods. The original sequence and its reverse complement log-likelihood scores were averaged to obtain the final log-likelihood values for both variant and reference sequences. The Delta likelihood scores were then computed by subtracting the reference log-likelihoods from the corresponding variant log-likelihoods.

To contextualize these findings, we performed the same analysis on variants annotated in the ClinVar database. Specifically, for each gene harboring a candidate or suggestive variant in our dataset (Tables 2–4; Supplementary Data 1 and Table S15), we retrieved all ClinVar entries classified as *pathogenic* or *likely pathogenic*, together with a randomly downsampled subset of *variants of uncertain significance* (VUS) and *benign/likely benign* (B/LB) variants. For genes with more than 450 annotated variants, B/LB and VUS variants were pruned to a total of 450 variants per gene (Supplementary Data 1 - Table S15).

ClinVar annotations were harmonized into three major categories: (i) *Pathogenic/likely pathogenic* (including “Pathogenic,” “Likely pathogenic,” and “Pathogenic/Likely pathogenic”); (ii) *Benign/likely benign* (including “Benign,” “Likely benign,” and “Benign/Likely benign”); and (iii) *Uncertain significance* (including “Uncertain significance” and “Conflicting interpretation of pathogenicity”). In total, 17,054 ClinVar variants (8255 Uncertain_significance; 6045 Benign/Likely_benign; 2754 Pathogenic/Likely_pathogenic) were analyzed using the Evo2 framework (Supplementary Data 1 and Table S15).

We then visualized the distribution of Evo 2 delta scores across these ClinVar categories and overlaid a dotted line indicating the distribution of delta scores for the variants identified in our study (Supplementary Data 1 and Tables S14 and S15), allowing direct comparison of our findings with known variant classifications in the same genes.

### Secondary findings

Although not directly related to the primary aim of our study, WGS analysis of this NDD cohort enabled the identification of several secondary findings associated with other clinically relevant and actionable conditions. We define as *secondary findings* variants classified as pathogenic or likely pathogenic by ClinVar, InterVar, or both. Variants were considered only if found in heterozygous state in an autosomal dominant gene, or in homozygous or hemizygous state in a recessive gene. The analysis was restricted to the 84 genes recommended by the American College of Medical Genetics and Genomics (ACMG) for clinical reporting^75^.

As part of the informed consent process designed for this study, participants were offered the option to receive information about any clinically relevant secondary findings. In accordance with ethical guidelines and data protection regulations, only those who explicitly opted in are contacted regarding individual results and informed about the need for further molecular analyses. Only secondary findings that strictly adhere to the reporting criteria outlined by the American College of Medical Genetics and Genomics (ACMG) are disclosed. For these participants, a second blood sample is collected, and diagnostic validation of the identified variant is conducted using standard clinical procedures. A formal report is then prepared, and individuals are referred for genetic counselling. A clinical geneticist will then disclose and explain the validated secondary findings to the participants or their legal guardians.

## Supplementary information

Supplementary Information

Supplementary Data 1

## Data Availability

The data supporting the results of this study are available on request from the corresponding author. The data are not publicly available due to privacy or ethical restrictions.
