# Supplementary material for: New insights into neurodevelopmental disorders by whole genome sequencing of 100 families from Italy
Source: NPJ Genom Med. 2026 Feb 2;11:11. doi: 10.1038/s41525-025-00547-8 (PMC12886966; doi:10.1038/s41525-025-00547-8)
Supplement: Supplementary file 1 — Supplementary Information [file 41525_2025_547_MOESM1_ESM.pdf]

Additional Information

Figure S1: comparison of Evo 2 delta scores between candidate and suggestive SNV/indels found in probands with variants annotated in the database ClinVar in the same genes. Plots were produced with R (ggplot2 package).

Figure S2: genomic read-depth coverage at loci overlapping candidate or suggestive CNVs.

Supplementary Data 1:
Table S1. Cohort overview;
Table S2. Kinship analysis;
Table S3. Quality checks (fastqc);
Table S4. Alignment metrics (samtools flagstat);
Table S5. Coverage metrics (samtools coverage);
Table S6. Filtered SNV/Indel (HaplotypeCaller);
Table S7. Filtered CNVs (CNVkit);
Table S8. Filtered CNVs (Vialle et al. Pipeline);
Table S9. Filtered insertions (Manta);
Table S10. Filtered MEI (Melt);
Table S11. Comparison between relevant variants and variants annotated in the denovo-db ssc database;
Table S12. Variant validation;
Table S13. Evidence supporting the involvement in neurodevelopment of genes affected by suggesting variants;
Table S14. Candidate and suggestive SNV/indel analysed with Evo 2;
Table S15. Variants annotated in the ClinVar database analysed with Evo 2;
Table S16. Genes of interest for NDDs.

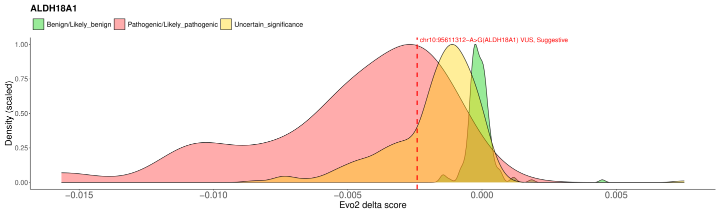
ALDH18A1
Benign/Likely_benign
Pathogenic/Likely_pathogenic
Uncertain_significance
chr10:95611312−A>G(ALDH18A1) VUS, Suggestive
Density (scaled)
Evo2 delta score

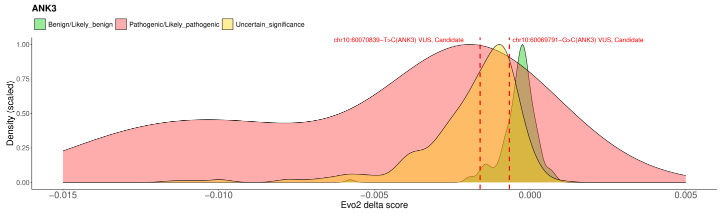
ANK3
Benign/Likely_benign
Pathogenic/Likely_pathogenic
Uncertain_significance
chr10:60070839−T>C(ANK3) VUS, Candidate
chr10:60069791−G>C(ANK3) VUS, Candidate
Density (scaled)
Evo2 delta score

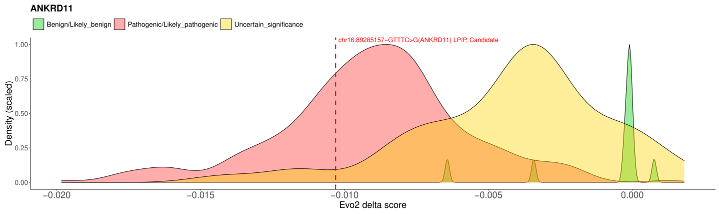
ANKRD11
Benign/Likely_benign
Pathogenic/Likely_pathogenic
Uncertain_significance
chr16:89285157−GTTTC>G(ANKRD11) LP/P, Candidate
Density (scaled)
Evo2 delta score

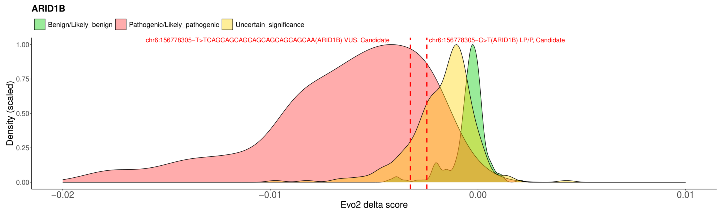
ARID1B
Benign/Likely_benign
Pathogenic/Likely_pathogenic
Uncertain_significance
chr6:156778305−T>TCAGCAGCAGCAGCAGCAGCAGCAA(ARID1B) VUS, Candidate
chr6:156778305−C>T(ARID1B) LP/P, Candidate
Density (scaled)
Evo2 delta score

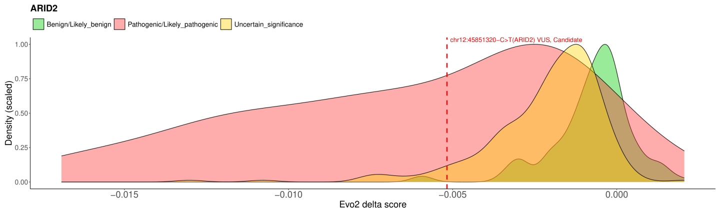
ARID2
Benign/Likely_benign
Pathogenic/Likely_pathogenic
Uncertain_significance
chr12:45851320−C>T(ARID2) VUS, Candidate
Density (scaled)
Evo2 delta score

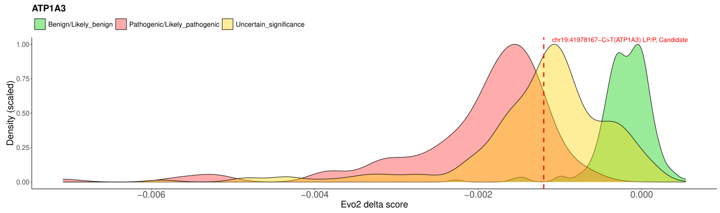
ATP1A3
Benign/Likely_benign
Pathogenic/Likely_pathogenic
Uncertain_significance
chr19:41978167−C>T(ATP1A3) LP/P, Candidate
Density (scaled)
Evo2 delta score

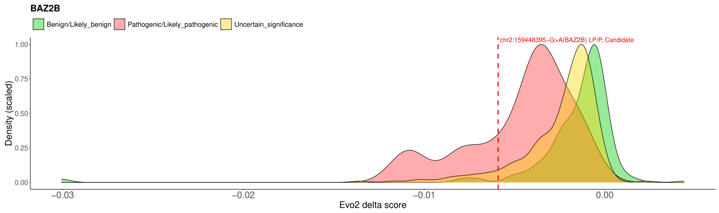
BAZ2B
Benign/Likely_benign
Pathogenic/Likely_pathogenic
Uncertain_significance
chr2:159448395−G>A(BAZ2B) LP/P, Candidate
Density (scaled)
Evo2 delta score

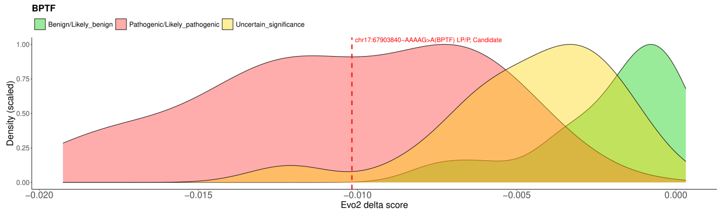
BPTF
Benign/Likely_benign
Pathogenic/Likely_pathogenic
Uncertain_significance
chr17:67903840−AAAAG>A(BPTF) LP/P, Candidate
Density (scaled)
Evo2 delta score

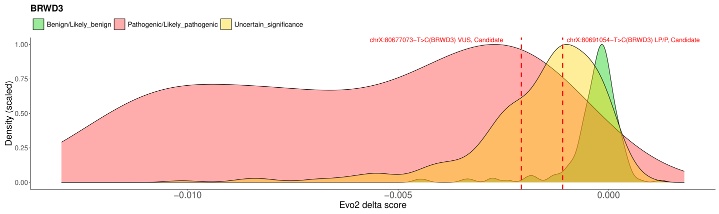
BRWD3
Benign/Likely_benign
Pathogenic/Likely_pathogenic
Uncertain_significance
chrX:80677073−T>C(BRWD3) VUS, Candidate
chrX:80691054−T>C(BRWD3) LP/P, Candidate
Density (scaled)
Evo2 delta score

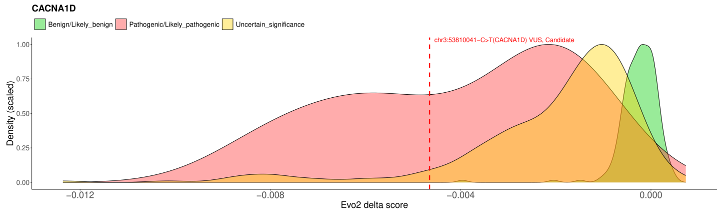
CACNA1D
Benign/Likely_benign
Pathogenic/Likely_pathogenic
Uncertain_significance
chr3:53810041−C>T(CACNA1D) VUS, Candidate
Density (scaled)
Evo2 delta score

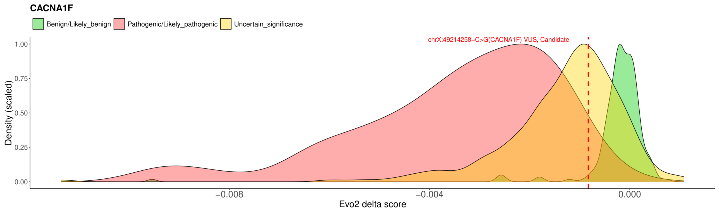
CACNA1F
Benign/Likely_benign
Pathogenic/Likely_pathogenic
Uncertain_significance
chrX:49214258−C>G(CACNA1F) VUS, Candidate
Density (scaled)
Evo2 delta score

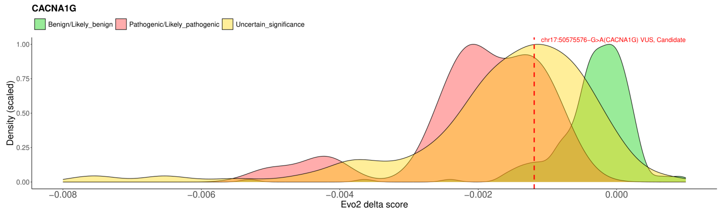
CACNA1G
Benign/Likely_benign
Pathogenic/Likely_pathogenic
Uncertain_significance
chr17:50575576−G>A(CACNA1G) VUS, Candidate
Density (scaled)
Evo2 delta score

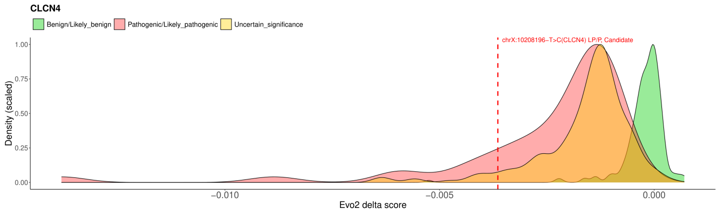
CLCN4
Benign/Likely_benign
Pathogenic/Likely_pathogenic
Uncertain_significance
chrX:10208196−T>C(CLCN4) LP/P, Candidate
Density (scaled)
Evo2 delta score

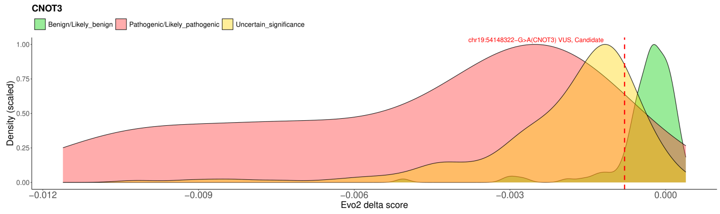
CNOT3
Benign/Likely_benign
Pathogenic/Likely_pathogenic
Uncertain_significance
chr19:54148322−G>A(CNOT3) VUS, Candidate
Density (scaled)
Evo2 delta score

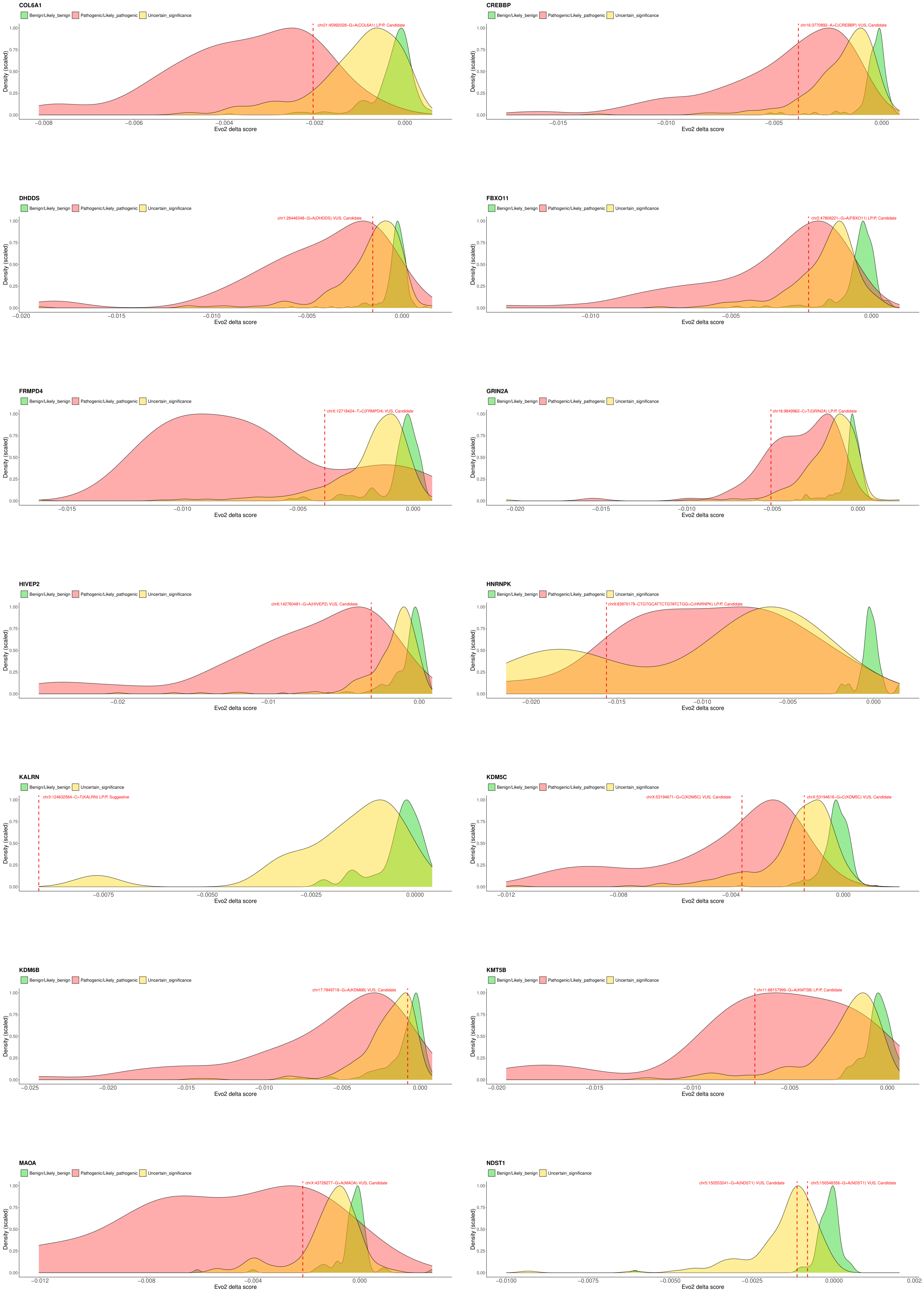
COL6A1
chr21:45992028−G>A(COL6A1) LP/P, Candidate
CREBBP
chr16:3770892−A>C(CREBBP) VUS, Candidate
DHDDS
chr1:26446348−G>A(DHDDS) VUS, Candidate
FBXO11
chr2:47808221−G>A(FBXO11) LP/P, Candidate
FRMPD4
chrX:12718424−T>C(FRMPD4) VUS, Candidate
GRIN2A
chr16:9849962−C>T(GRIN2A) LP/P, Candidate
HIVEP2
chr6:142760481−G>A(HIVEP2) VUS, Candidate
HNRNPK
chr9:83970179−CTGTGCATTCTGTATCTGG>C(HNRNPK) LP/P, Candidate
KALRN
chr3:124632564−C>T(KALRN) LP/P, Suggestive
KDM5C
chrX:53194671−G>C(KDM5C) VUS, Candidate
chrX:53194616−G>C(KDM5C) VUS, Candidate
KDM6B
chr17:7849719−G>A(KDM6B) VUS, Candidate
KMT5B
chr11:68157999−G>A(KMT5B) LP/P, Candidate
MAOA
chrX:43728277−G>A(MAOA) VUS, Candidate
NDST1
chr5:150553241−G>A(NDST1) VUS, Candidate
chr5:150548356−G>A(NDST1) VUS, Candidate
Benign/Likely_benign
Pathogenic/Likely_pathogenic
Uncertain_significance
Density (scaled)
Evo2 delta score

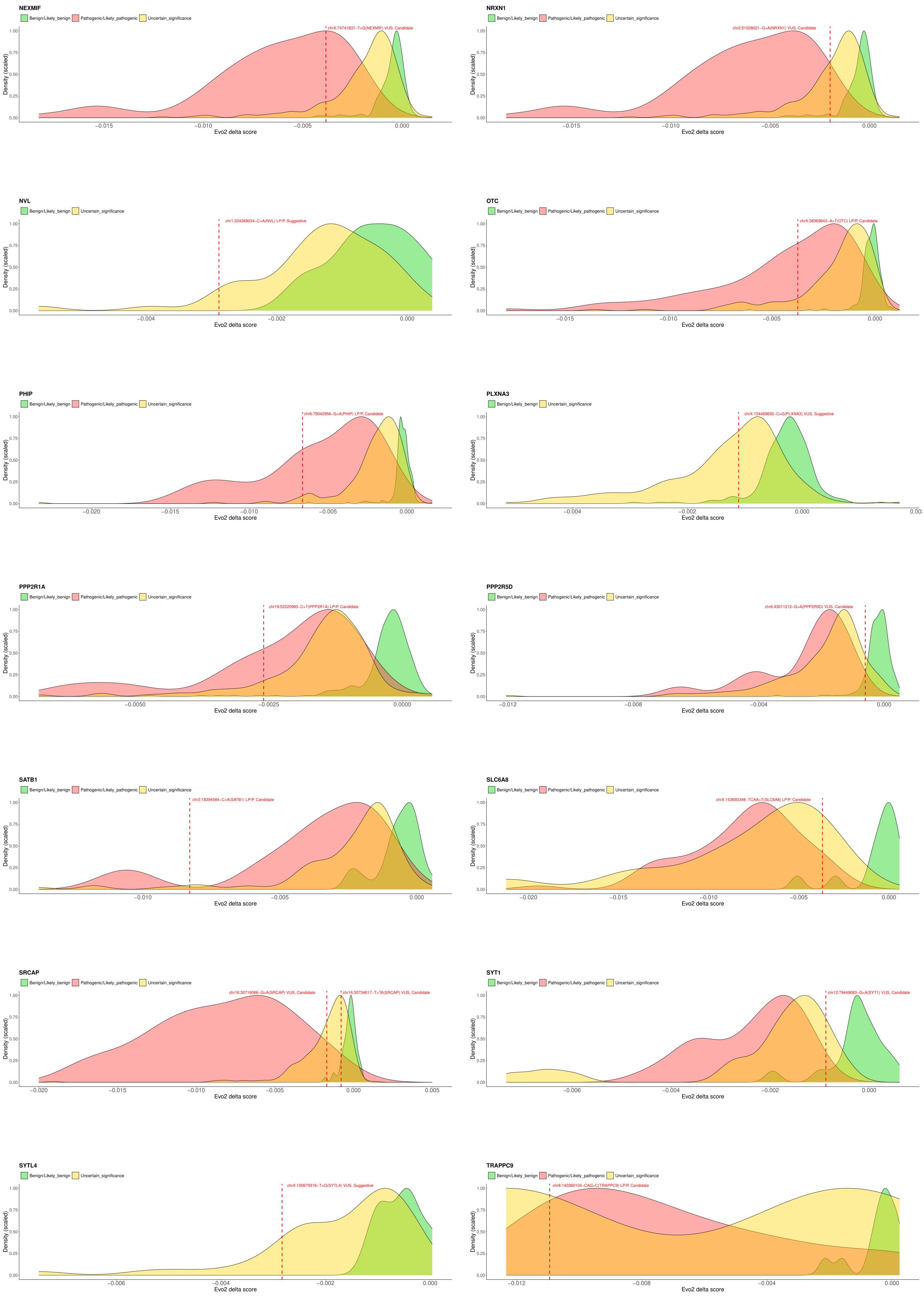
NEXMIF
Benign/Likely_benign
Pathogenic/Likely_pathogenic
Uncertain_significance
chrX:74741837–T>G(NEXMIF) VUS, Candidate
Density (scaled)
Evo2 delta score
NRXN1
chr2:51028021–G>A(NRXN1) VUS, Candidate
NVL
chr1:224268034–C>A(NVL) LP/P, Suggestive
OTC
chrX:38369843–A>T(OTC) LP/P, Candidate
PHIP
chr6:79042956–G>A(PHIP) LP/P, Candidate
PLXNA3
chrX:154469692–C>G(PLXNA3) VUS, Suggestive
PPP2R1A
chr19:52220990–C>T(PPP2R1A) LP/P, Candidate
PPP2R5D
chr6:43011212–G>A(PPP2R5D) VUS, Candidate
SATB1
chr3:18394584–C>A(SATB1) LP/P, Candidate
SLC6A8
chrX:153693348–TCAA>T(SLC6A8) LP/P, Candidate
SRCAP
chr16:30716066–G>A(SRCAP) VUS, Candidate
chr16:30734617–T>TA(SRCAP) VUS, Candidate
SYT1
chr12:79449083–G>A(SYT1) VUS, Candidate
SYTL4
chrX:100679316–T>G(SYTL4) VUS, Suggestive
TRAPPC9
chr8:140360104–CAG>C(TRAPPC9) LP/P, Candidate

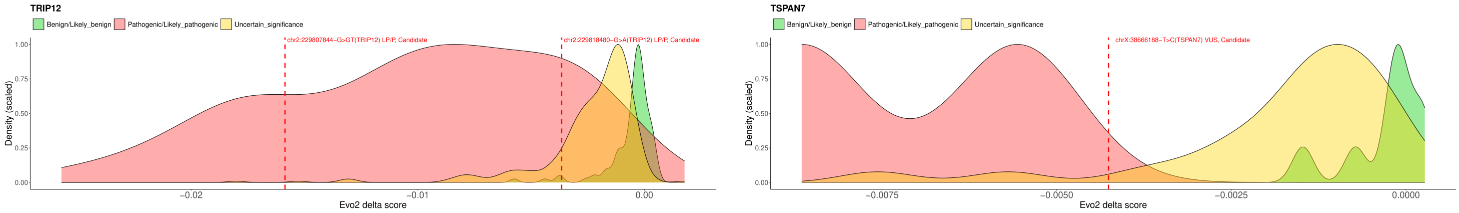

Figure S1: comparison of Evo 2 delta scores between candidate and suggestive SNV/indels found in probands with variants annotated in the database ClinVar in the same genes. Plots were produced with R (ggplot2 package).

a.

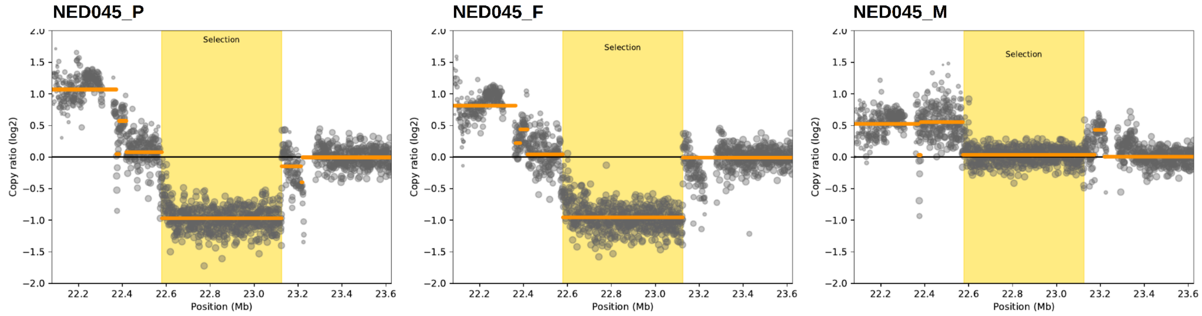
NED045_P
NED045_F
NED045_M
Selection
Copy ratio (log2)
Position (Mb)
22.2 22.4 22.6 22.8 23.0 23.2 23.4 23.6

b.

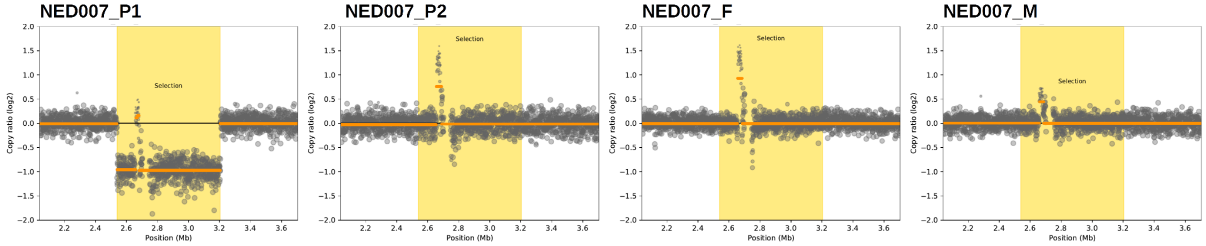
NED007_P1
NED007_P2
NED007_F
NED007_M
Selection
Copy ratio (log2)
Position (Mb)
2.2 2.4 2.6 2.8 3.0 3.2 3.4 3.6

c.

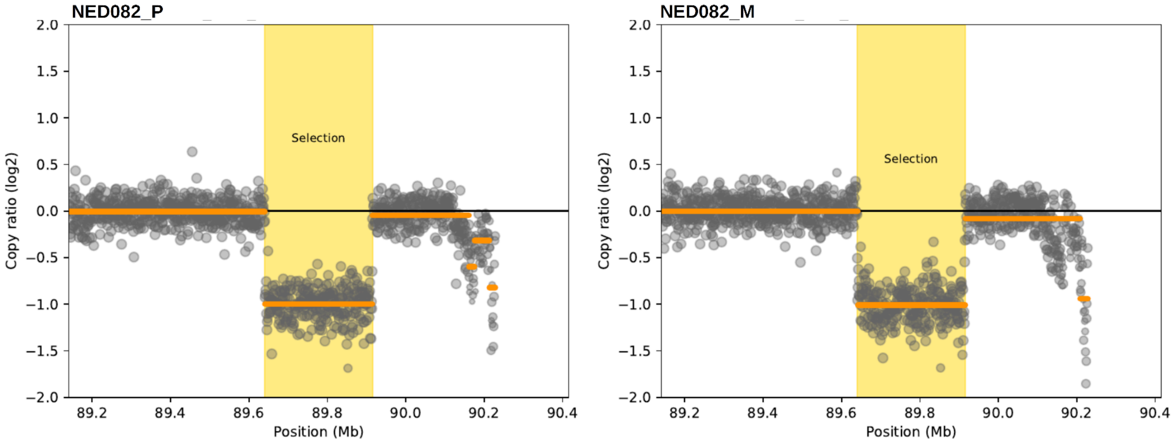
NED082_P
NED082_M
Selection
Copy ratio (log2)
Position (Mb)
89.2 89.4 89.6 89.8 90.0 90.2 90.4

d.

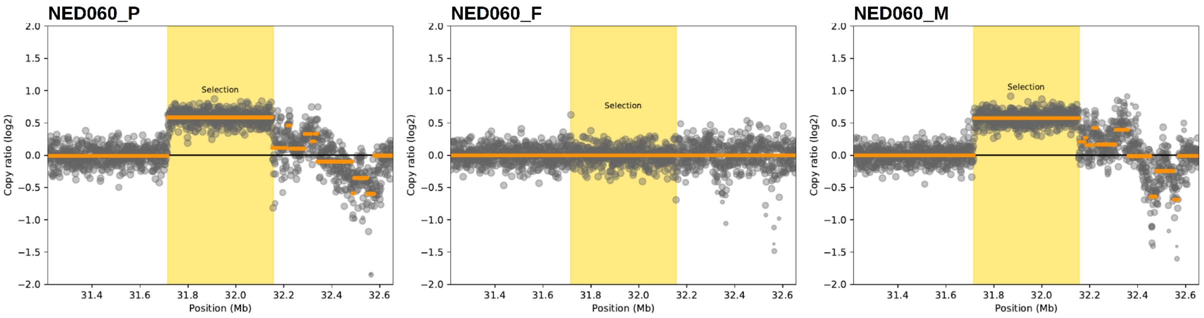
NED060_P
NED060_F
NED060_M
Selection
Copy ratio (log2)
Position (Mb)
31.4 31.6 31.8 32.0 32.2 32.4 32.6

e.

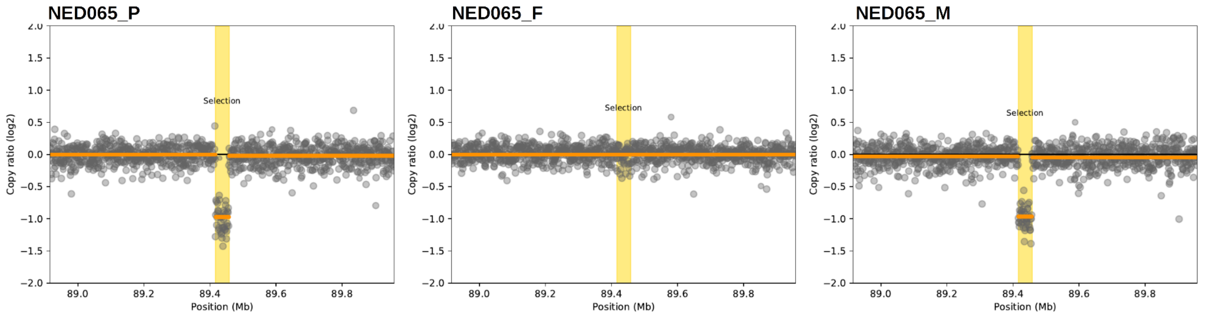

NED065_P
NED065_F
NED065_M
Selection
Copy ratio (log2)
Position (Mb)

f.

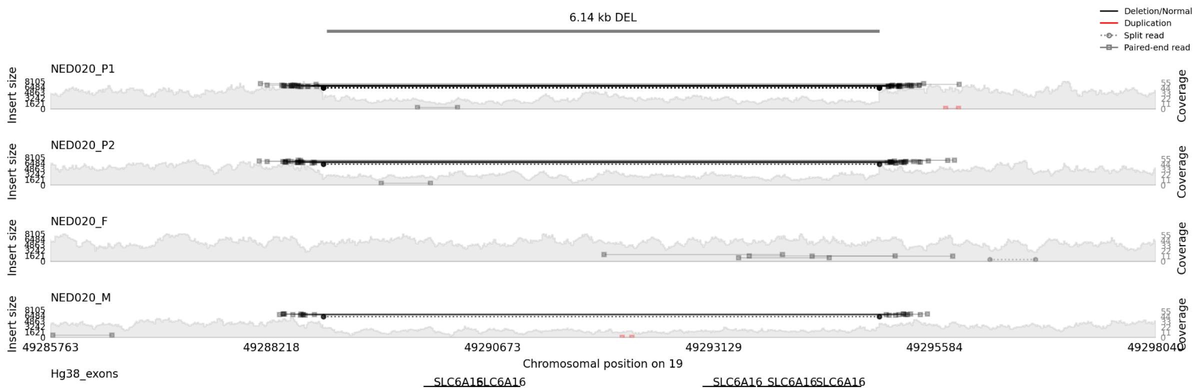

6.14 kb DEL
Deletion/Normal
Duplication
Split read
Paired-end read
NED020_P1
NED020_P2
NED020_F
NED020_M
Insert size
Coverage
49285763
49288218
49290673
49293129
49295584
49298040
Chromosomal position on 19
Hg38_exons
SLC6A16

g.

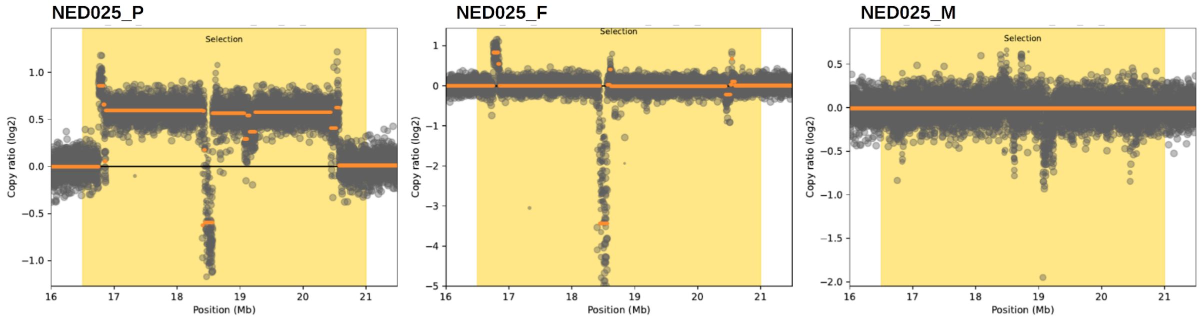

NED025_P
NED025_F
NED025_M
Selection
Copy ratio (log2)
Position (Mb)

h.

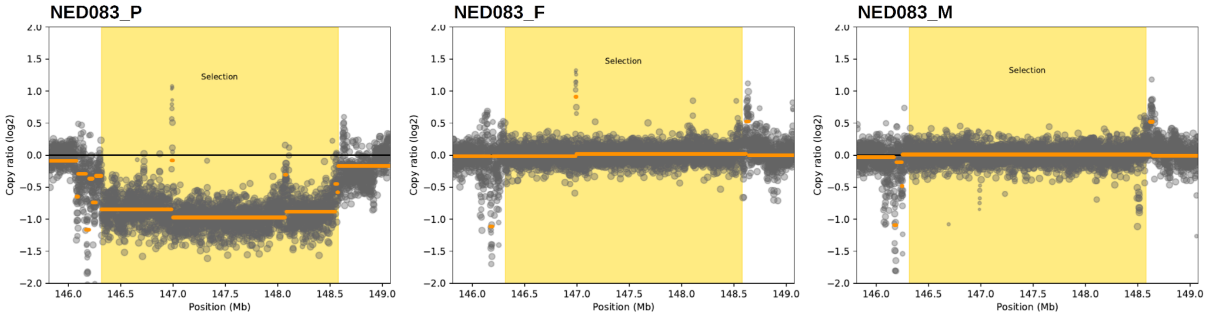
NED083_P
NED083_F
NED083_M
Selection
Copy ratio (log2)
Position (Mb)

i.

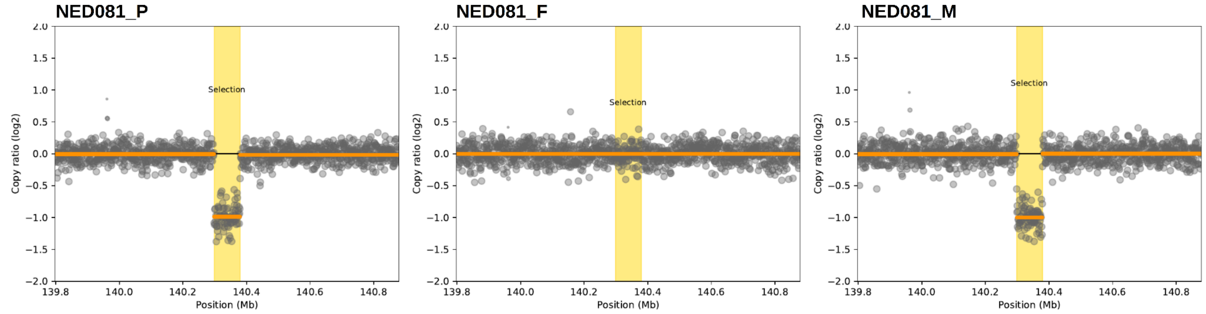
NED081_P
NED081_F
NED081_M
Selection
Copy ratio (log2)
Position (Mb)

j.

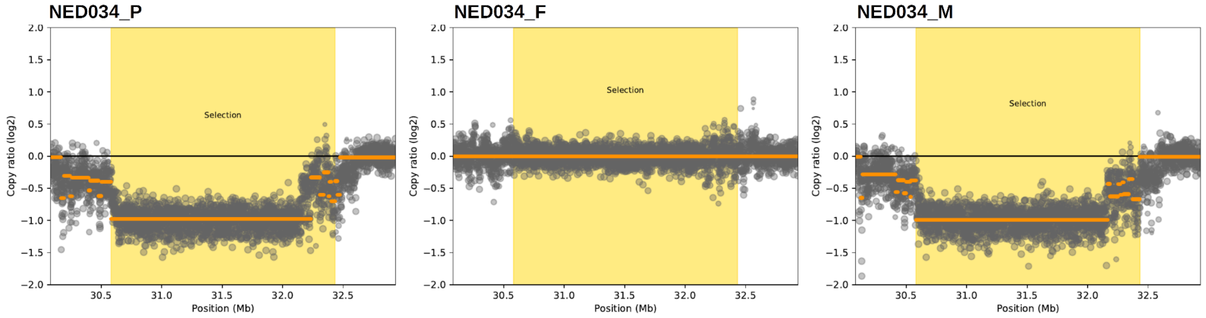
NED034_P
NED034_F
NED034_M
Selection
Copy ratio (log2)
Position (Mb)

k.

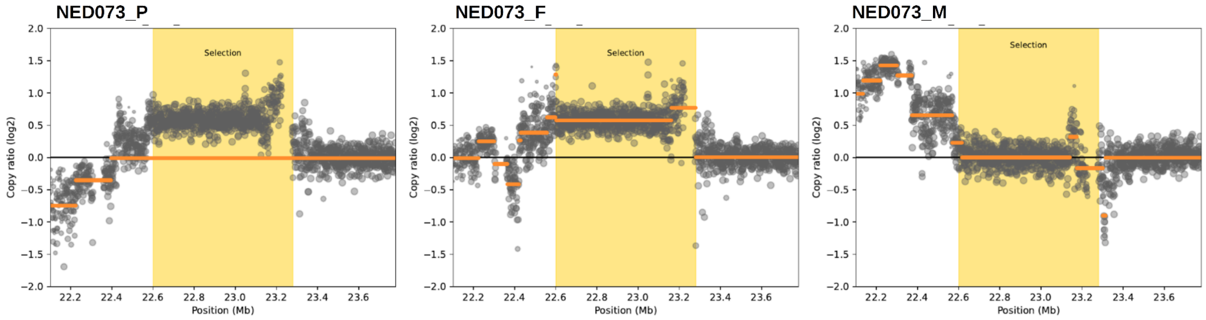
NED073_P
NED073_F
NED073_M
Selection
Copy ratio (log2)
Position (Mb)

l.

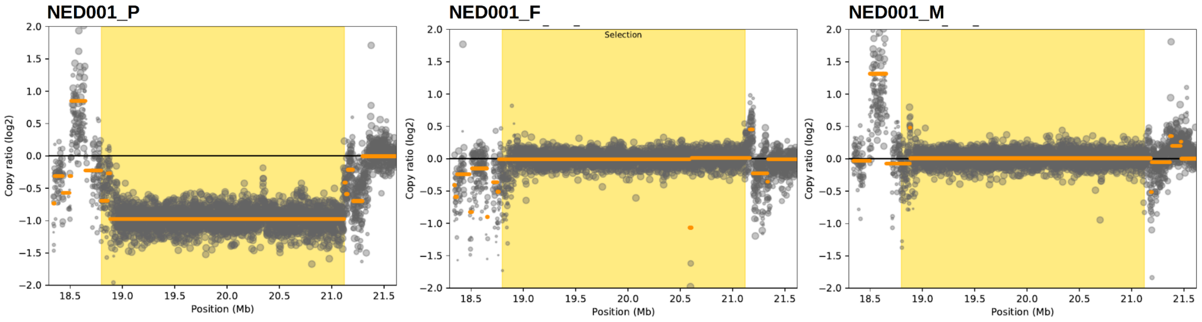
NED001_P
NED001_F
NED001_M
Selection
Copy ratio (log2)
Position (Mb)

m.

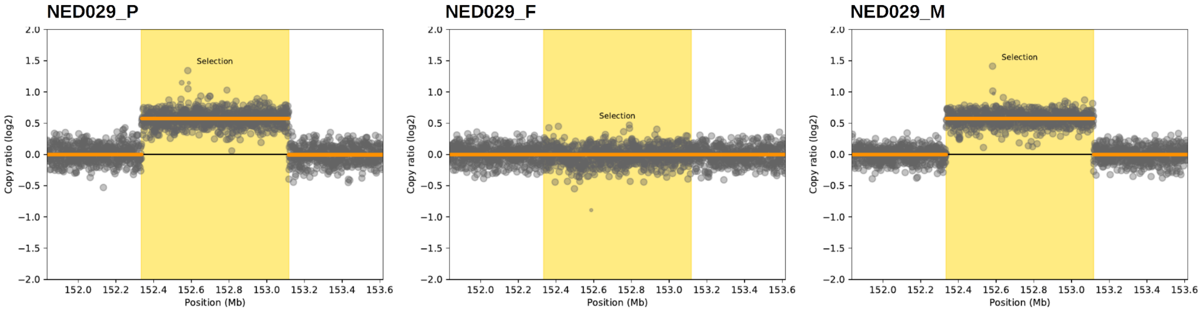
NED029_P
NED029_F
NED029_M
Selection
Copy ratio (log2)
Position (Mb)

n.

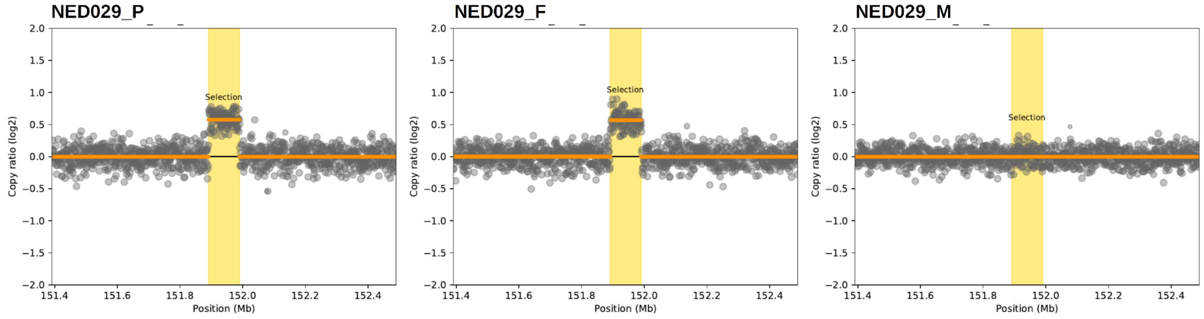
NED029_P
NED029_F
NED029_M
Selection
Copy ratio (log2)
Position (Mb)

o.

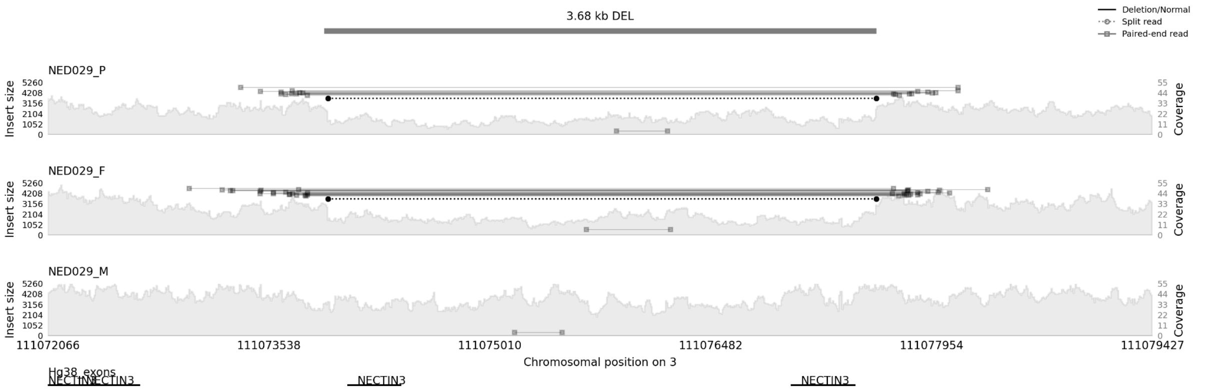
3.68 kb DEL
Deletion/Normal
Split read
Paired-end read
NED029_P
NED029_F
NED029_M
Insert size
Coverage
111072066
111073538
111075010
111076482
111077954
111079427
Chromosomal position on 3
Hg38_exons
NECTIN3
NECTIN3
NECTIN3

p.

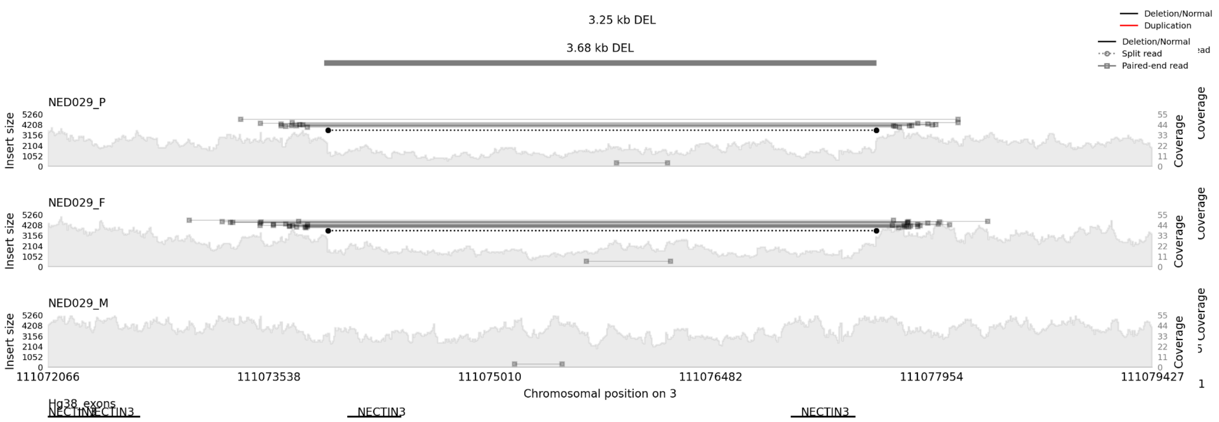
3.25 kb DEL
3.68 kb DEL
Deletion/Normal
Duplication
Deletion/Normal
Split read
Paired-end read
NED029_P
NED029_F
NED029_M
Insert size
Coverage
111072066
111073538
111075010
111076482
111077954
111079427
Chromosomal position on 3
Hg38_exons
NECTIN3
NECTIN3
NECTIN3

q.

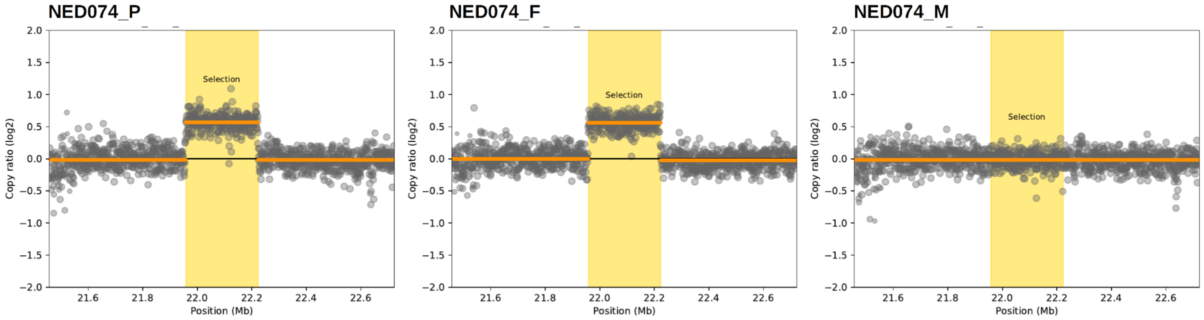
NED074_P
NED074_F
NED074_M
Selection
Copy ratio (log2)
Position (Mb)

r.

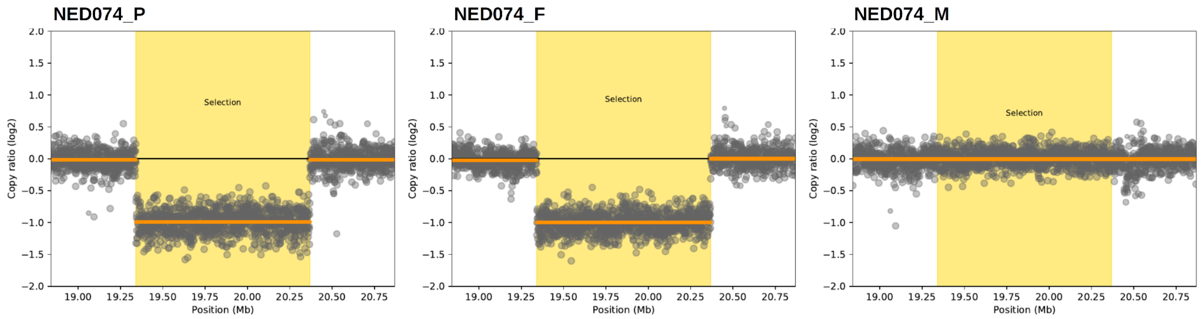
NED074_P
NED074_F
NED074_M
Selection
Copy ratio (log2)
Position (Mb)

s.

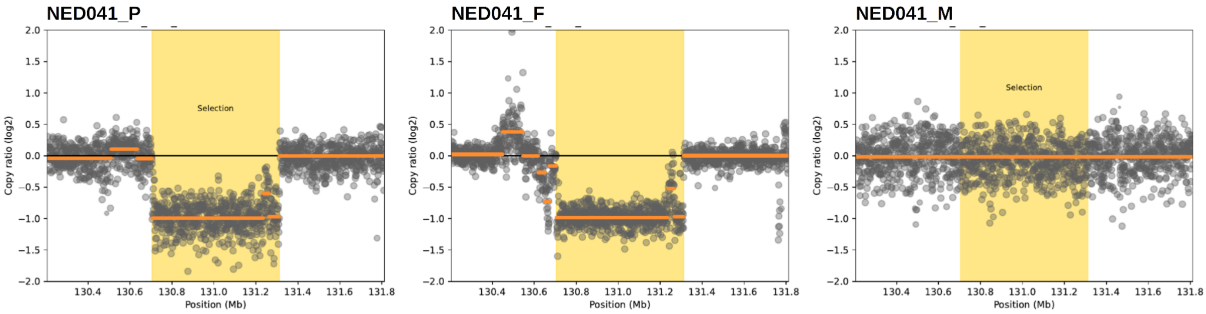
NED041_P
NED041_F
NED041_M
Selection
Copy ratio (log2)
Position (Mb)

t.

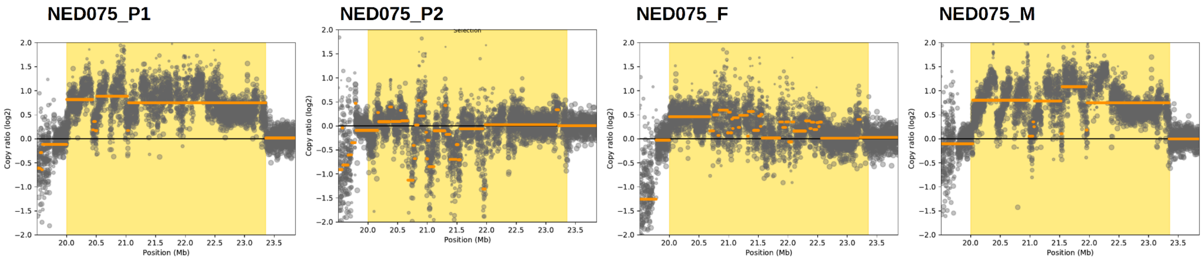
NED075_P1
NED075_P2
NED075_F
NED075_M
Copy ratio (log2)
Position (Mb)

u.

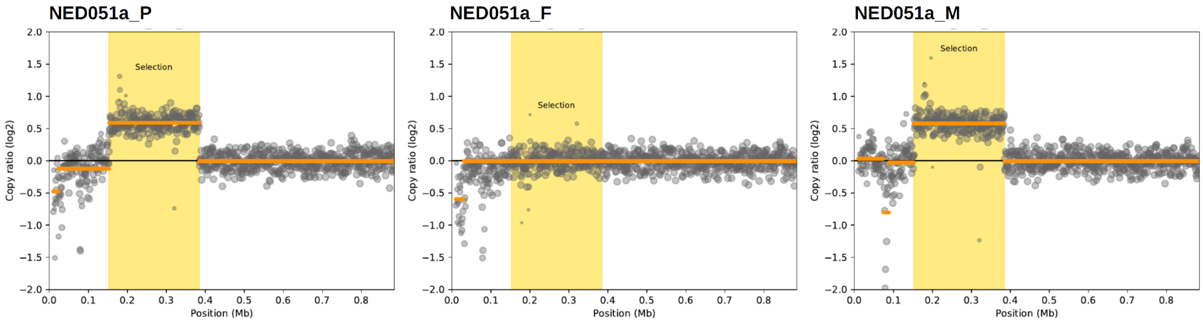
NED051a_P
NED051a_F
NED051a_M
Selection
Copy ratio (log2)
Position (Mb)

Figure S2 - Genomic read-depth coverage at loci overlapping candidate or suggestive CNVs: a. Representation of genomic coverage at the site of the deletion NC_000015.10:g.22579193_23125407del detected in the father and the proband of family NED045 through bioinformatic analysis using CNVkit; **b.** Representation of genomic coverage at the site of the deletion NC_000001.11:g.2538784_3205123del detected in the proband (NED007_P1) of family NED007 through bioinformatic analysis using CNVkit; **c.** Representation of genomic coverage at the site of the deletion NC_000016.10:g.89640587_89914558del detected in the proband and the mother of family NED082 through bioinformatic analysis using CNVkit; **d.** Representation of genomic coverage at the site of the duplication NC_000015.10:g.31714997_32155815dup detected in the proband and the mother of family NED060 through bioinformatic analysis using CNVkit; **e.** Representation of genomic coverage at the site of the deletion NC_000016.10:g.89415912_89457624del detected in the proband and the mother of family NED065 through bioinformatic analysis using CNVkit; **f.** Representation of genomic coverage at the site of the deletion NC_000019.10:g.49288832_49294971del detected in both probands and the mother of family NED020 through bioinformatic analysis using Vialle *et al.* pipeline for the detection of structural variants; **g.** Representation of genomic coverage at the site of the duplication NC_000017.11:g.16751633_20572952dup detected in the proband of family NED025 through bioinformatic analysis using CNVkit; **h.** Representation of genomic coverage at the site of the deletion NC_000001.11:g.146169841_148577763del detected in the proband of family NED083 through bioinformatic analysis using CNVkit; **i.** Representation of genomic coverage at the site of the deletion NC_000008.11:g.140297895_140379423del detected in the proband and the mother of family NED081 through bioinformatic analysis using Vialle *et al.* pipeline for the detection of structural variants; **j.** Representation of genomic coverage at the site of the deletion NC_000015.10:g.30581192_32433578del detected in the proband an the mother of family NED034 through bioinformatic analysis using CNVkit; **k.** Representation of genomic coverage at the site of the duplication NC_000015.10:g.20019885_23284457dup detected in the proband an the father of family NED073 through bioinformatic analysis using CNVkit; **l.** Representation of genomic coverage at the site of the deletion NC_000022.11:g.18751100_21330400del detected in the proband of family NED001 through bioinformatic analysis using CNVkit; **m.** Representation of genomic coverage at the site of the duplication NC_000007.14:g.152335080_153117170dup detected in the proband and the mother of family NED029 through bioinformatic analysis using CNVkit; **n.** Representation of genomic coverage at the site of the duplication NC_000005.10:g.151891111_151989703dup detected in the proband and the father of family NED029 through bioinformatic analysis using CNVkit; **o.** Representation of genomic coverage at the site of the deletion NC_000019.10:g.47034087_47037339del detected in the proband and the father of family NED029 through bioinformatic analysis using Vialle *et al.* pipeline for the detection of structural variants; **p.** Representation of genomic coverage at the site of the deletion NC_000003.12:g.111073906_111077587del detected in the proband and the father of family NED029 through bioinformatic analysis using the bioinformatic pipeline we implemented for the detection of structural variants; **q.** Representation of genomic coverage at the site of the duplication NC_000022.11:g.21957373_22222810dup detected in the proband and the father of family NED074 through bioinformatic analysis using CNVkit; **r.** Representation of genomic coverage at the site of the deletion NC_000017.11:g.19340574_20369135del detected in the proband and the father of family NED074 through bioinformatic analysis using CNVkit; **s.** Representation of genomic coverage at the site of the deletion NC_000002.12:g.130702734_131311349del detected in the proband and the father of family NED041 through bioinformatic analysis using CNVkit; **t.** Representation of genomic coverage at the site of the duplication NC_000015.10:g.20000921_23357453dup detected in the proband (NED075_P1) and the mother of family NED075 through bioinformatic analysis using CNVkit; **u.** Representation of genomic coverage at the site of the duplication NC_000009.12:g.152201_384463dup

detected in the proband and the father of family NED051b through bioinformatic analysis using CNVkit. All plots were produced either with CNVkit or Samplot.
